# Characterization of Oncogenic and Immunogenic Profiling in Patients with Breast Cancer Tumors After Radiation Therapy

**DOI:** 10.3390/ijms27073227

**Published:** 2026-04-02

**Authors:** Suryakant Niture, Carlos E. Vargas, Saranya Chumsri, Jennifer M. Kachergus, Sandeepkumar Sriramanujam, Dinesh Thotala, Jerry Jaboin, Danushka Seneviratne

**Affiliations:** 1 Department of Radiation Oncology, Oklahoma University Health Sciences Center, Oklahoma City, OK 73104, USA; 2 Stephenson Cancer Center, Oklahoma University, Oklahoma City, OK 73104, USA; 3 Department of Radiation Oncology, Mayo Clinic Comprehensive Cancer Center, Jacksonville, FL 32224, USA

**Keywords:** gene biomarker, breast cancer, oncogene, immune cells, residual cancer burden

## Abstract

Biological heterogeneity among different breast cancer (BC) subtypes results in markedly varying clinical outcomes. Identification and analysis of key gene biomarkers that are differentially regulated during radiation therapy (RT) may pose multiple clinical challenges for BC treatment. The purpose of the study is to identify and analyze the expression of key gene biomarkers and their networks that are differentially regulated after hypofractionated RT. Patients with BC (cT0-T2, N0, M0) were treated with hypofractionated whole breast RT 25 Gy in five fractions, 4 to 8 weeks before breast conservation surgery (BCS). Biopsy (pre-RT; *n* = 5) and surgical (post-RT; *n* = 14 or 15) BC tumor samples were used for NanoString targeted sequencing. We identified 165 and 244 differentially expressed genes (DEGs; *p* < 0.05) in BC tumor samples from BC patients post-RT using the nCounter BC360 and IO360 panels, respectively. Gene networks and pathway analysis revealed that RT increases the gene signature of tumor inflammation (TIS), cytotoxicity, and apoptosis, while downregulating the gene signatures of tumor cell proliferation, differentiation, and cell adhesion, and increases the claudin-low gene score. RT-induced mammary stemness and enhanced infiltration of stroma, mast, and macrophage cells in the BC tumor microenvironment (TME). Further, the nCounter IO360 (immuno-oncology) panel analysis validated the findings of BC360 and demonstrated that RT increased the myeloid inflammation signature and chemokine expression, modulated B, T, NK, and DC cell activities, and enhanced residual cancer burden (RCB) in BC tumors, thus creating an immunosuppressive TME. Collectively, RT sensitized BC tumors by increasing the gene signature of TIS, cytotoxicity, apoptosis, and mammary stemness. RT facilitated an immunosuppressive environment and increased RCB, suggesting that the therapeutic potential of RT is highly individualized for each patient based on their unique tumor biology, genetic makeup, and TME.

## 1. Introduction

Breast cancer (BC) remains the second leading cause of cancer death among women worldwide, and about one in eight women is diagnosed (13%) with BC in the United States [1]. In 2025, about 316,950 invasive and 59,080 non-invasive BC cases, with 42,170 women’s deaths, are estimated to occur in the United States (https://seer.cancer.gov/statfacts/html/breast.html; accessed on 12 December 2025). Aside from chemotherapy, surgery, and immunotherapy, radiation therapy (RT) is believed to improve locoregional control of BC [2,3]. RT enhances systemic responses to cancer immunotherapy and is used in adjuvant, neoadjuvant, and palliative treatments and in managing secondary BC [4,5,6,7]. RT improves the systemic response to cancer immunotherapy [8,9,10]. RT induces DNA breaks and micronucleus formation, activating nucleic acid sensors in the cytoplasm, which can trigger the cyclic GMP-AMP synthase–stimulator of interferon genes (cGAS-STING) pathway, leading to the expression of type I interferon (IFN-I) [11]. Additionally, RT upregulates FAS (a death receptor) and MHC class I and translocates calreticulin to tumor cell surfaces, while also increasing HMGB1 release from dying tumor cells, collectively inducing tumor cell death [12]. In addition, RT induces immunogenic cell death (ICD) as a functional immune response against BC, which converts the BC tumor into an in situ vaccine. ICD releases tumor antigens that regulate pro-inflammatory signals (Interferon-β, HMGB1, and ATP), and promotes mature dendritic cell (DC) and cytotoxic CD8+ T cell infiltration into the TME and tumor [10,13,14]. However, radioresistance is common in BC because of tumor heterogeneity/subtypes, genetic mutations, the presence of cancer stem cells, dysregulated DNA repair mechanisms, cell cycle alterations, oncogenic signaling variations, and immune modulation in the BC-TEM [15,16]. The molecular factors influencing these differing treatment outcomes remain poorly understood, necessitating further research to discover novel drug targets, especially for aggressive BC subtypes.

Here, we utilized BC360 and IO360 panels for biomarker discovery to predict the pathological complete responses (pCR) after RT in BC tumors directly [17,18]. The gene expression data obtained from NanoString nCounter BC-specific BC360 panel and immunology panel IO360 were analyzed from biopsy samples vs. post-RT samples comparatively. RT’s impact on inflammatory and oncogenic signaling, immune cells regulation, and overall immune response-associated pathways with the BC-TME was analyzed. This study leverages biomarker discovery for a prognostic model of BC in feature selection.

## 2. Results

### 2.1. BC Tumor Characterization

**Figure 1:**
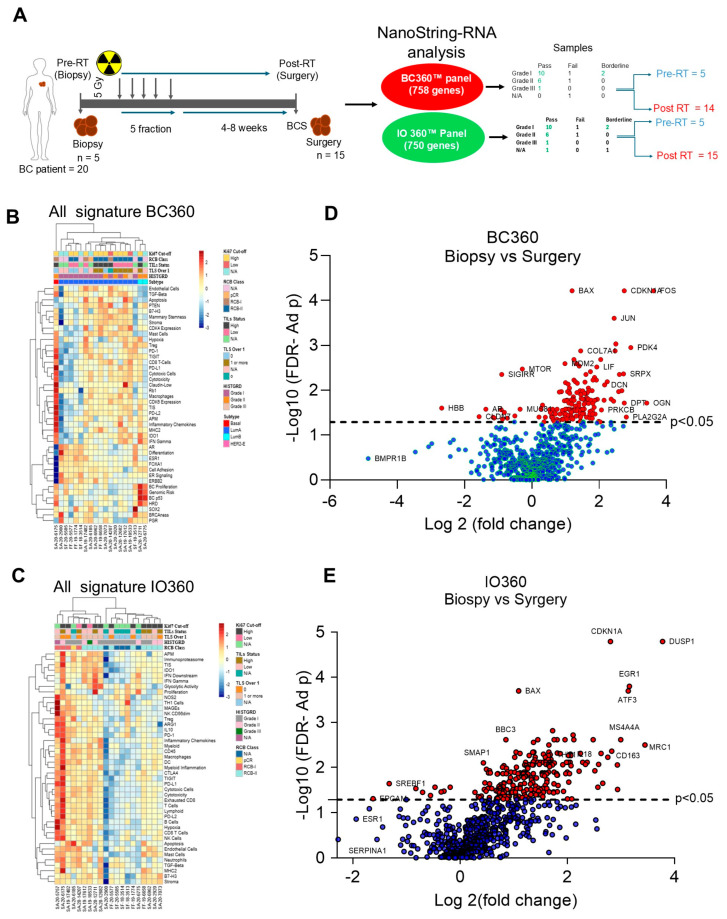
**BC tumor characterization and differential gene expression (DGE) after post-RT.** (**A**) A schematic represents the cohort study design, RT strategy, and RNA sample analysis by NanoString BC360 and IO360 panels. The detailed procedure is described in the Materials and Methods section. (**B**) Heat map representing tumor characterization (BC tumor subtypes, histological grade, RCB class, Ki67 cut-off, and TIL status of biopsy (pre-RT; *n* = 5) and surgical (post-RT *n* = 14) analyzed by BC360 panel. (**C**) Heat map representing tumor characterization and immune pathway analysis pre-RT (*n* = 5) and post-RT (*n* = 15) analyzed by IO360 panel. (**D**,**E**) Differential gene expression (DGE) analyses of the BC360 and IO360 panels were performed. Gene expression (fold change) was determined, with *p*-values adjusted within each analysis, using a difference *t*-test followed by the Benjamini and Yekutieli False Discovery Rate (FDR) adjustment to account for correlations amongst the tests, and statistical magnitude and significance were determined. Significantly adjusted (*p* < 0.05) cut-off expression key genes are presented (red circles) and labeled.

Between 2019 and 2020, we enrolled 20 patients of different ages with varying stages and grades of BC in this cohort study, as described in Table S1 and Figure 1A. Tumor characterization reveals that 16 tumors belong to LumA, 2 tumors belong to LumB, and 1 tumor belongs to the basal BC subtype. Histologically, 10 tumors are classified as grade I, 6 tumors as grade II, and 1 tumor as grade III. We collected a biopsy specimen from 5 patients before RT and after RT from 15 patients’ surgical samples. We used the BC360 (nCounter Breast Cancer 360) panel to analyze 19 samples (5 biopsy and 14 surgical; QC passed). For the panCancer IO360 panel, we used 20 patient samples (5 biopsy and 15 surgical: QC passed) (Figure 1A). Comparisons within the report include HISTGRD, TLS (0 vs. 1+), TIL Status (Low <= 10% vs. High >= 10%), Ki67 Analysis (Low <= 1% vs. High = 1% or more), and RCB Class (RCB-I vs. RCB-II or N/A-pCR), Time Series Analysis for all samples, and matched samples (Figure 1A).

### 2.2. Characterization of Gene Expression

Comparative nCounter analysis (*t*-test followed by FDR analysis) as presented in Figure S1A,B suggests that the BC360 and IO360 panels identified 758 and 750 differentially expressed genes (DEGs), respectively. Interestingly, BC360 and IO360 gene expression reveals that specifically 100 and 179 genes (*p* < 0.05) have changed their expression, respectively, whereas 65 up- and downregulated common genes were found in both analyses after RT (Figure S2A, upper panel). This analysis prompted us to explore the impact of RT on targeted gene biomarker identification for further significance analysis. All signatures of BC360 and IO360 panels (heat maps) revealed that about ~43 pathways/factors (including tumor signaling, tumor cell-associated pathways, and immune invasion) are differentially expressed in each patient tumor (Figure 1B,C). Heat maps of BC360 and IO360 further suggest that basal BC tumors (1) and residual cancer burden class I (RCB I) tumors (4) show high aggressiveness by modulation of the inflammatory response (Figure 1B,C), whereas LumB-BC subtype tumors show high cell proliferation activity, genomic risk, and HRD activities compared with LumA-BC subtype tumors (Figure 1B, Table S1). BC360 panCancer comparative gene regulation analysis of biopsy vs. surgery tumors revealed that several genes, including *FOS*, *CDKN1A*, *BAX*, *THY1*, *JUN*, *COL7A1*, *HLA-DPA1*, *MTOR*, *FSTL1*, *VIM*, *ITGB1*, *MMP14*, *DPT*, and others, were significantly upregulated. In contrast, genes such as *HBB*, *AR*, *CLDN1*, *SIDIRR*, and others were downregulated (Figure 1D and Figure S2B). On the other hand, IO360 analysis revealed that *DUSP1*, *ATF3*, *EGR1*, *MRC*, *MS4A4A*, *MRC1*, *CD163*, *IL10RA*, *CDKN1A*, *BAX*, and others were significantly upregulated (*p* < 0.05) post-RT compared with biopsy samples (Figure 1E and Figure S2C). This data clearly suggests that RT induces several key gene expressions in BC tumors, which participate in BC tumor modulation and may be associated with BC patients’ prognosis.

### 2.3. RT Increased BRCAness and Modulated Immune Cell Activity

Because RT damages DNA primarily, radiation and BRCAness have a complex relationship associated with *BRCA1/2* gene mutations and impaired DNA repair. Radiation does not create a *BRCA* mutation; it can unmask or induce a “BRCAness” phenotype in cells with impaired DNA repair mechanisms [19]. To examine RT’s impact on BRCAness, we analyzed comparative gene biomarker expression, and our data suggest that RT increased the expression of *DCN* (Decorin) 4.13-fold, *HLA-E* 2.14-fold, *GJB2* 1.52-fold, *BTG2* 1.79-fold, and *CD68* 2.82-fold. Increased expression of these genes significantly decreased BRCAness. No significant change in expression of *BRCA1*, *BRCA2*, and homologous recombination deficiency (HRD) related to *RAD51* and *RAD51C* genes was observed in biopsy vs. surgical BC tumors (Figure S3A,B). Overall BRCAness score was significantly downregulated, whereas HRD score did not considerably change in biopsy vs. RT-exposed tumors (Figure S3A,B). Since the genomic risk associated with HRD and BRCAness plays an important role in tumor development and therapeutic responses, we further characterize the genomic risk score. Gene expression overall data suggest downregulated genomic risk (*p* = 0.064) in RT-exposed tumors compared with the biopsy tumor (Figure S3C). Moreover, we comparatively analyzed the genomic risk of four patients’ biopsy samples (not exposed to RT) vs. the same tumors after RT, and the data show a high variation in genomic risk score, and overall downregulation of genomic risk (*p* < 0.62) (Figure S3D). In addition, gene expression analysis suggests that RT does not significantly change the score of ERS and p53 signaling (Figure S4A,B, upper and lower panels). However, RT significantly increased AR, RB1 signaling, CDK4, CDK6, B7-H3, IDO1, PD-L2, CD8-T cell, MHCII machinery, macrophage, and mast cell scores, and no change in the APM score was observed (Figures S5A–C and S6A,B). The overall heat maps, comparative gene expression analysis, and score determination suggest that RT downregulates BRCAness and modulates genomic instability in BC tumors as expected.

### 2.4. RT Suppresses Cell Proliferation/Differentiation and Increases the Claudin-Low Score in BC Tumors

**Figure 2:**
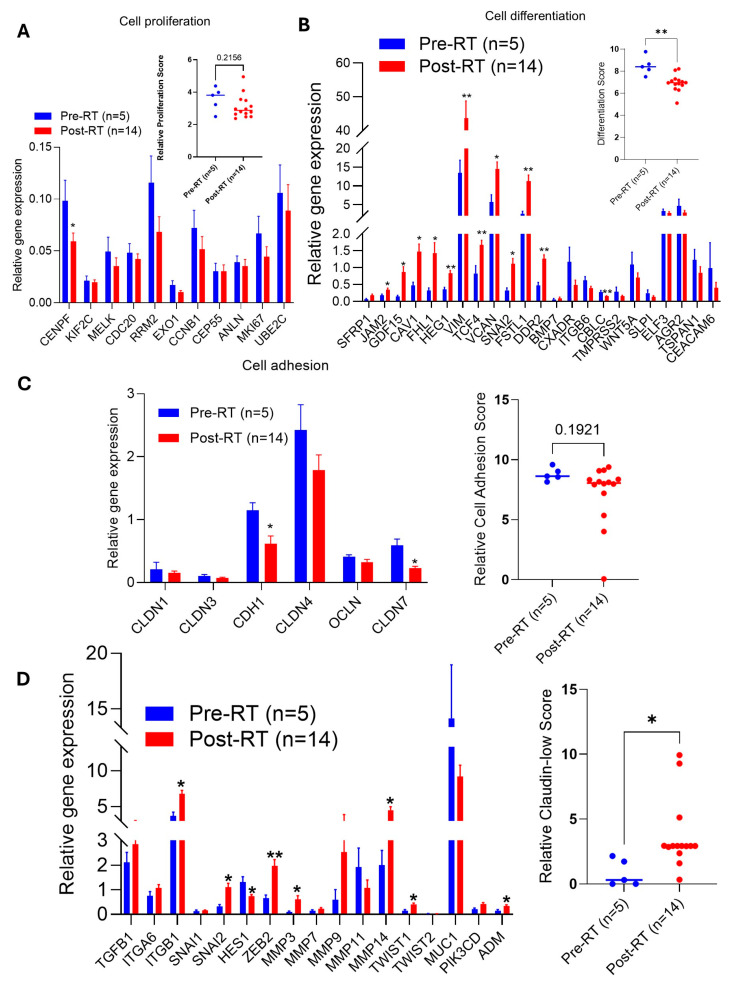
**Effect of RT on BC tumor cell proliferation, differentiation, and cell adhesion**. (**A**–**D**) Comparative gene expressions (BC360: biopsy *n* = 5 vs. surgery *n* = 14) associated with tumor cell proliferation (**A**), tumor cell differentiation (**B**), cell adhesion (**C**), and claudin-low-related (**D**) genes were analyzed using the BC360 panel. The relative score of each pathway was calculated as described in the Materials and Methods section. * *p*  <  0.05, and ** *p*  <  0.01 compared with biopsy tumor samples.

We analyzed the impact of RT on tumor cell proliferation, differentiation, and cell adhesion after RT. Most of the genes related to cell proliferation, such as *CENPF* (*p* < 0.05), *MELK*, *RRM2*, *CCNB1*, *MKI67*, and *UBE2C*, are downregulated, and the proliferation score also decreased by 26.8% compared to biopsy tumor samples (Figure 2A and inset graph). In addition, RT significantly increased expression of *JAM2* (2.02-fold), *GDF15* (5.97-fold), *CAV1* (3.14-fold), *FHL1* (4.49-fold), *HEG1* (2.37-fold), *VIM* (3.24-fold), *TCF4* (2.03-fold), *VCAN* (2.57-fold), *SNAI2/SLUG* (3.43-fold), *FSTL1* (4.34-fold) and *DDR2* (2.73-fold) (Figure 2B). In contrast, the expression of *CBLC* was significantly downregulated (0.53-fold) in RT treated tumors, whereas cell differentiation was decreased by 20% (*p* < 0.01) in post-RT tumors (Figure 2B and inset graph).

Claudins are transmembrane proteins that are critical for cell adhesion at tight junctions (TJs); however, their dysregulation can affect cell proliferation, migration, and metastasis by activating matrix metalloproteinases (MMPases) [20,21]. We analyze the expressions of four claudins, including *CLDN1*, *CLDN3*, *CLDN4*, and *CLDN7*, as well as *CDH1* (E-cadherin regulator) and *OCLN* (tight junction regulator). Claudin expression was downregulated in post-RT BC tumors, specifically *CLDN7* (0.372-fold; significant), whereas *CDH1* (0.372-fold; significant) was also downregulated; however, the overall cell adhesion score was not significantly (*p* = 0.19) downregulated in biopsy vs. surgical (post-RT) tumors (Figure 2C). Moreover, in post-RT samples, the expression of *ITGB1* (1.83-fold), *SNAI2/SLUG* (3.43-fold), *ZEB2* (3.03-fold), *MMP3*, and *MMP14* (6.1- and 2.26-fold), *TWIST2* (2.66-fold), and *ADM* (2.26-fold) was significantly upregulated, leading to an increased (~20%; *p* < 0.05) claudin-low score in post-RT samples compared with biopsy samples (Figure 2D). Collectively, this data indicates that RT increased the claudin-low score that signifies poor prognosis and aggressive BC subtypes.

### 2.5. RT Induces TIS, Cytotoxicity, and Apoptosis

**Figure 3:**
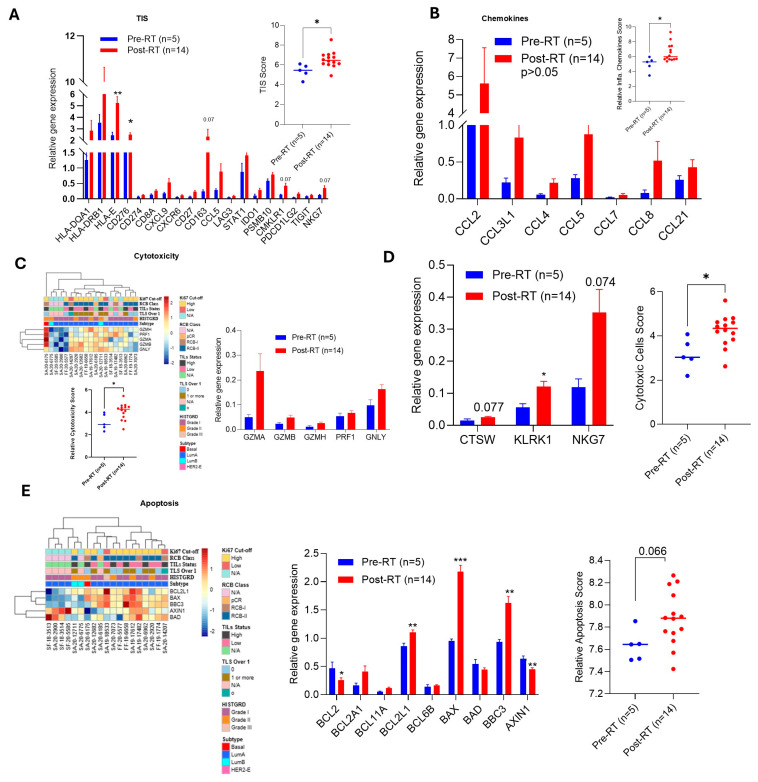
**RT upregulated tumor inflammation signature (TIS)-associated genes and induced cytotoxicity and apoptosis**. Using the BC360 panel analysis, comparative gene expression (biopsy *n* = 5 vs. surgery *n* = 14) associated with TIS and TIS score (inset graph) was determined (**A**), and relative chemokine genes and score (inset graph) were determined (**B**). The RT effect on BC tumor cell cytotoxicity and cytotoxic cell score was calculated after BC360 panel analysis (**C**,**D**), and the RT effect on tumor cell apoptosis-related gene expression and score was analyzed after BC360 panel analysis (**E**). * *p*  <  0.05, ** *p*  <  0.01 and *** *p*  <  0.01 compared with gene expression of biopsy tumor samples.

The tumor inflammation signature (TIS) is an 18-transcriptomic biomarker, based on an 18-gene signature, that measures the presence of a pre-existing adaptive immune response within a tumor [22]. Our data suggests that compared to biopsy tumor samples, post-RT surgical BC tumors showed a high and significant TIS score (*p* < 0.05) (Figure 3A and inset graph). The cause of increased TIS after RT was the increased MHC class I/II and immune cell gene expression. RT increased expression of MHC class II genes, such as *HLA-DQA1* (2.24-fold; *p* = 0.23), *HLA-DRB1* (2.48-fold), MHC class I gene *HLA-E* (2.16-fold), *CD276* (B7-H3) immune checkpoint protein (1.42-fold), *CD274* (*PD-L1*; 1.64-fold), *PDCD1LG2* (3.42-fold), *CD8A* (1.94-fold), T and B cell-specific *CD27* (3.14-fold), M2 macrophage marker *CD163* (9.59 fold), immune modulator *STAT1* (1.62-fold), natural killer (NK) cells and CD8 T cell-associated gene *NKG7* (2.95-fold), suggesting RT increased TIS in BC TME (Figure 3A and inset graph). RT also increased chemokine gene expression, such as *CXCL9*, *CXCR6*, *CCL5*, *CMKLR1*, *CCL2, CCL3L1*, *CCL4*, *CCL5*, *CCL7*, *CCL8*, and *CCL21* (not significantly), indicating different types of lymphoid and myeloid inflammatory responses (Figure 3B and inset graph). NK and T cells related to *GZMA*, *GZMB*, *GZMH*, *PRF1*, and *GNLY* genes are upregulated in RT-exposed BC tumors; therefore, the overall cytotoxicity score is significantly high (2.27-fold) in post-RT surgical tumors (Figure 3C, all three panels). The infiltration of NK and CD8+ T cells in the BC-TME increased significantly because of the expression of *CTSW* (1.63-fold), *KLRK1* (2.16-fold), and *NKG7* genes with a high cytotoxic cell score of 2.61-fold (*p* < 0.05) (Figure 3D, left and right panels). Additionally, RT induced the expression of *BBC3* (*PUMA*: 1.73-fold) and *BAX* (2.27-fold), pro-apoptotic genes, significantly, as well as downregulating the anti-apoptotic breast cancer prognostic marker gene *BCL2* (0.55-fold) and cell fate and Wnt signaling modulator gene *AXIN1* (0.710-fold), leading to an increased overall apoptosis score (*p* = 0.06) (Figure 3E, all three panels). Collectively, our data suggested that RT enhanced TIS, cytotoxicity, cytotoxic cell score, and cell apoptosis in BC tumors.

### 2.6. RT Enhances Mammary Stemness, Stromal, and Endothelial Cell Score in BC-TME

**Figure 4:**
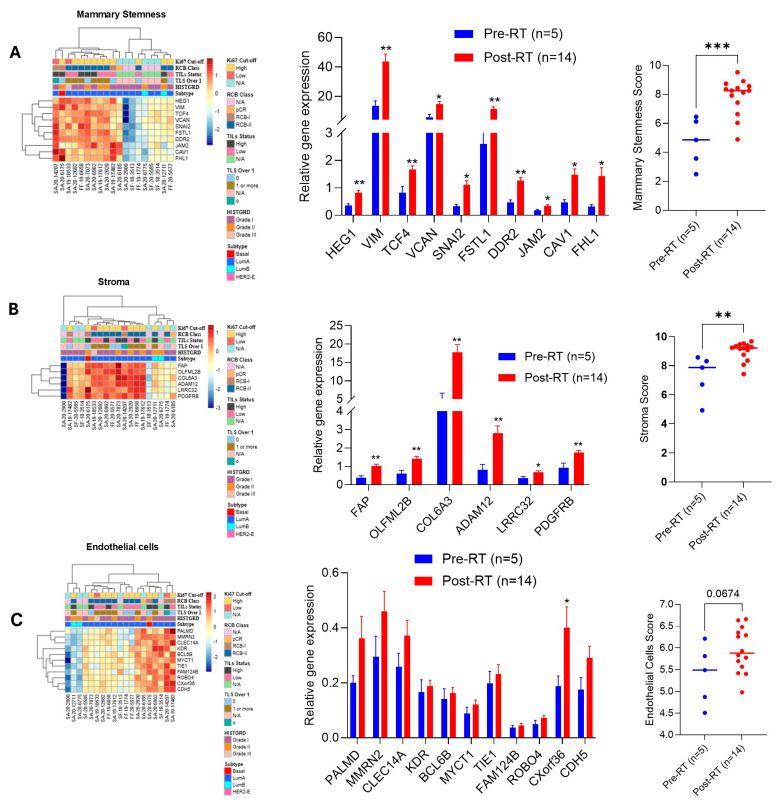
**RT upregulated gene expression related to the mammary stemness and stroma and endothelial cells in the BC tumor milieu**. (**A**) Impact of RT on mammary stemness-associated gene expression and mammary stemness score (**A**) were analyzed using the BC360 panel (biopsy *n* = 5 vs. surgery *n* = 14). (**B**,**C**) Effect of RT on stroma cell- and endothelial cell-associated gene expression; the score was calculated after BC360 panel analysis and plotted. The heap map represents each patient’s gene expression levels, and the bar graph represents the collective gene expression profile with statistical significance. * *p*  <  0.05, ** *p*  <  0.01, and *** *p*  <  0.01 compared with biopsy tumor samples.

Mammary stemness is an inherent ability for self-renewal, proliferation, and differentiation in normal and tumor cells, and several genes modulate this process [23]; our data analyzed several gene biomarkers related to BC stemness after RT in BC tumors. The increase in the expression of *HEG1* (2.36-fold), *VIM* (3.23-fold), *TCF4* (2.01-fold), *VCAN* (2.57-fold), *SNAI2* (3.41-fold), *FSTL1* (3.33-fold), *DDR2* (2.71-fold), *JAM1* (1.93-fold), *CAV1* (3.12-fold) and *FHL1* (4.49 fold) with overall mammary stemness score is 3.14-fold higher in post-RT BC tumors compared to biopsy samples (Figure 4A, left and middle panels). The stroma cell-associated biomarker expression, such as *FAB* (2.69-fold), *OLFML2B* (2.33-fold), *COL6A3* (3.58-fold), *ADAM12* (3.48-fold), *LRRC32* (1.91-fold), and *PDGFRB* (1.87-fold), increased significantly with the stroma cell score 3.16-fold after RT (Figure 4B, all three panels). In post-RT tumors, increasing stemness and stroma cell biomarker expression obviously invites endothelial cells into the BC-TME for angiogenesis [24], and data indicated that overall, the endothelial cell score was increased 1.50-fold (*p* = 0.067) (Figure 4C, all three panels), indicating that RT enhances mammary stemness and infiltration of stromal and endothelial cells into the BC-TME.

Overall, as presented in Figure S7A–C, BC360 gene expression revealed that RT increased several gene expressions, increased cell cytotoxicity and anti-inflammatory TME activity, and modulated residual cancer burden (RCB). In addition, biological/clinical scores suggest that RT sensitized basal BC subtypes (aggressive BC) predominantly compared with LumB and LumA BC subtypes (Figure S7D,E). These data suggest that RT not only sensitizes tumor cells for death but also modulates radioresistance in BC tumors by enhancing stemness and by attracting stromal and endothelial cells into the TME.

### 2.7. RT Upregulated TIS by Lymphoid Cell Activity in BC-TME (IO360 Analysis)

**Figure 5:**
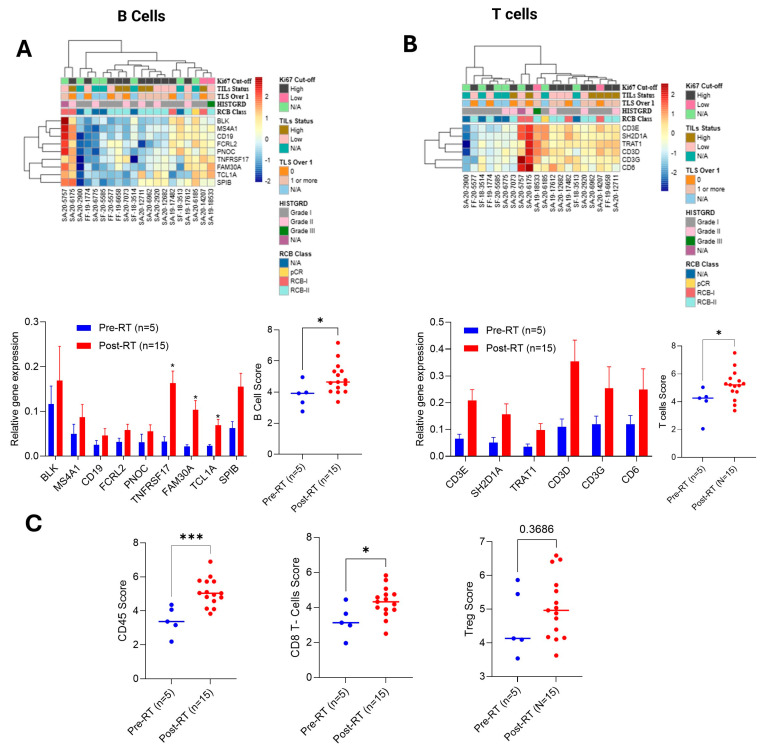
**RT regulates lymphoid B and T cell activity in BC-TME (IO360 analysis)**. (**A**,**B**) Upper panels: heat map represents the expression of B and T cell-specific gene markers in biopsy (*n* = 5) and surgery (*n* = 15) samples and their association with BC tumor grades and characteristics. Lower panels: B and T cell-specific gene expressions were plotted using the IO360 RNA nCounter data, and cell scores were analyzed and plotted. (**C**) Effect of RT on CD45 score, CD8 T cell score, and Treg score was determined comparatively (biopsy vs. surgery) and plotted. * *p*  <  0.05, and *** *p*  <  0.01 (*n* = 15) related to biopsy tumor samples (*n* = 5).

IO360 gene expression analysis further validates BC360 gene expression data; RT increased the tumor inflammation signature (TIS) score, cytotoxicity, and apoptotic gene expression signature significantly, as well as suppressing cell proliferation (Figures S8A,B and S9A–C, all panels). Using IO360 panels, we comparatively analyzed the lymphoid-related 56 gene expression from biopsy and surgical samples (Figure S10A). RT increased the overall lymphoid score by 27% (significantly) in the BC tumor (Figure S10A) and regulated a lymphoid-related cluster of differentiation genes (CDs), chemokines, granzymes, and transcription factor genes (Figure S10B–F). Furthermore, we specifically examined the level of expression of B cell and T cell-specific or common B cell and T cell genes after post-RT. The heat map indicates that B and T cell activities were associated with residual cancer burden class I (RCB I) and RCB class II level tumors, and most of the B and T cell-related gene markers were upregulated after post-RT (Figure 5A,B, upper and lower panels). Precisely, B cell-specific expression of *TNFRSF17* increased 4.977-fold, *FAM30A* 4.69-fold, and *TCL1A* 3.001-fold compared to biopsy samples, and overall B score increased in BC tumors 2.29-fold in post-RT BC tumor samples (*p* < 0.05). Although T cell-specific gene markers were not significantly upregulated, an increased expression of *CD3E*, *CD3D*, *CD3G*, *CD6*, *SH2D1A*, and *TRAT1* was observed upon RT, with an overall T cell score 2.61-fold higher in post-RT tumor samples (Figure 5B, lower panels). Moreover, the overall increasing CD45 score (B and T cell activator) (*p*< 0.001), CD8 T cell score (*p* < 0.05), and Treg score (*p* = 0.36) (Figure 5C) suggest that RT modulates B- and T-mediated anti-tumorigenic immune response in BC tumors.

### 2.8. RT Regulates Myeloid Activity in BC-TME and MHC Class II (MHCII) Gene Expression

**Figure 6:**
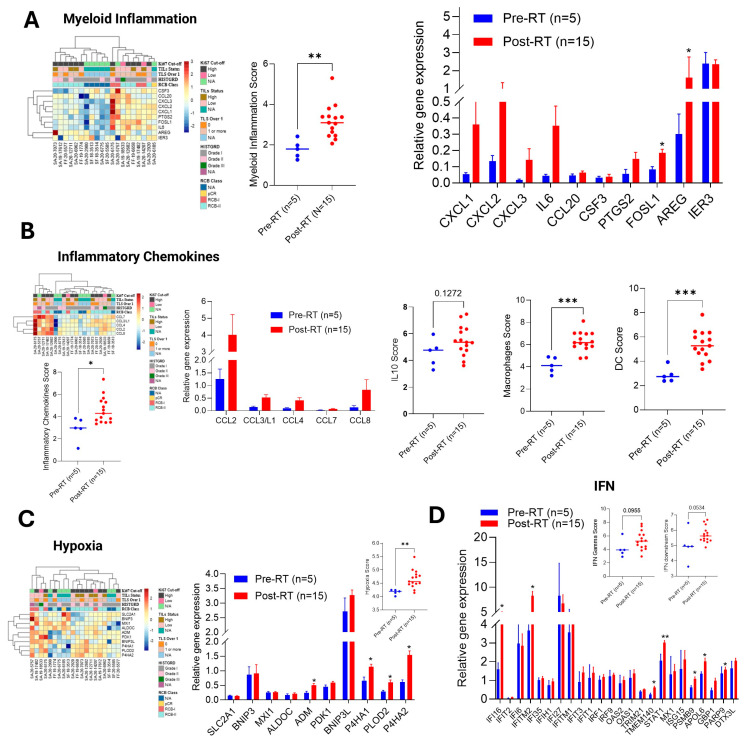
**RT increased myeloid inflammation, macrophages, DC cell score, and tumor hypoxia**. (**A**–**C**) The heat map represents DGEs of myeloid inflammation, inflammatory chemokines, and hypoxia in BC tumors before and after RT (left panels), and the relative gene expression and overall scores are presented (right panels). (**D**) The effect of RT on interferon signaling-related gene expression and overall interferon gene score is presented. * *p*  <  0.05, ** *p*  <  0.01, and *** *p*  <  0.01 related to biopsy tumor samples (*n* = 5).

We analyzed 37 myeloid-associated gene expressions after RT treatment in BC tumors, and the overall myeloid score significantly increased in post-RT surgical samples (*p* < 0.01) (Figure S11A–D), suggesting that RT enhanced myeloid cell activity in BC-TME predominantly (overall fold gene expression pattern) compared to lymphoid cells (Figures S10 and S11). To support this, we further analyzed myeloid-associated inflammatory signaling in post-RT tumors; most of the post-RT tumors’ myeloid inflammatory scores were significantly higher (*p* < 0.01) compared to biopsy tumor samples (Figure 6A, left and right panels). The pro-inflammatory chemokines *CXCL1*, *CXCL2*, *CXCL3*, and *IL6* are upregulated, and *FOSL1* and *AREG* genes are also significantly upregulated in post-RT samples (Figure 6A, right panel). Interestingly, macrophage-associated chemokine genes, such as *CCL2*, *CCL3*, *CCL4*, *CCL7*, and *CCL8*, are upregulated in post-RT-treated tumors (Figure 6B, upper and lower panels), with a significant overall chemokine score (*p* < 0.05). The expression of *IL-10* and macrophage and dendritic cell scores were significantly higher in post-RT tumors, suggesting that RT enhanced myeloid cell activity, particularly macrophages and DC, in BC-TEM (Figure 6B, right panels). In addition, our data suggests that RT induced BC tumor hypoxia significantly, and there was no significant change in interferon signaling observed (Figure 6C,D and inset graphs).

Indeed, because hypoxic and anti-inflammatory signaling associated with tumor-associated macrophages, particularly M2 macrophages and TAM-M2-associated *CD163* expression, represented one of the top upregulated genes in post-RT surgical tumors, this may create an anti-inflammatory environment that is known to promote tumor growth/survival and reduce the anti-tumor immune response and resistance to RT and immunotherapy [25]. Our overall data suggest that RT increased the immunosuppressive TME in BC tumors. Moreover, as demonstrated in Figures S12A–C and S13A,B, RT significantly increased the score of MHC2 machinery (*CTLA4*, *PD-L1*, *PD-L2*, *B7-H3*, *IDO1*), immunoproteasome, exhausted CD8 cells, stroma cells, mast cells, and dysregulated AMP scores. Since RT causes a complex series of changes in the TME, leading to increased numbers and altered functions of mast cells, endothelial cells, and stromal cells, our data suggest that an increasing population of mast cells, endothelial cells, and stromal cells in the BC-TME can promote tumor recurrence and therapy resistance.

### 2.9. RT Suppresses Immune Profiling with Increasing Residual Cancer Burden (RCB) in BC Tumors

**Figure 7:**
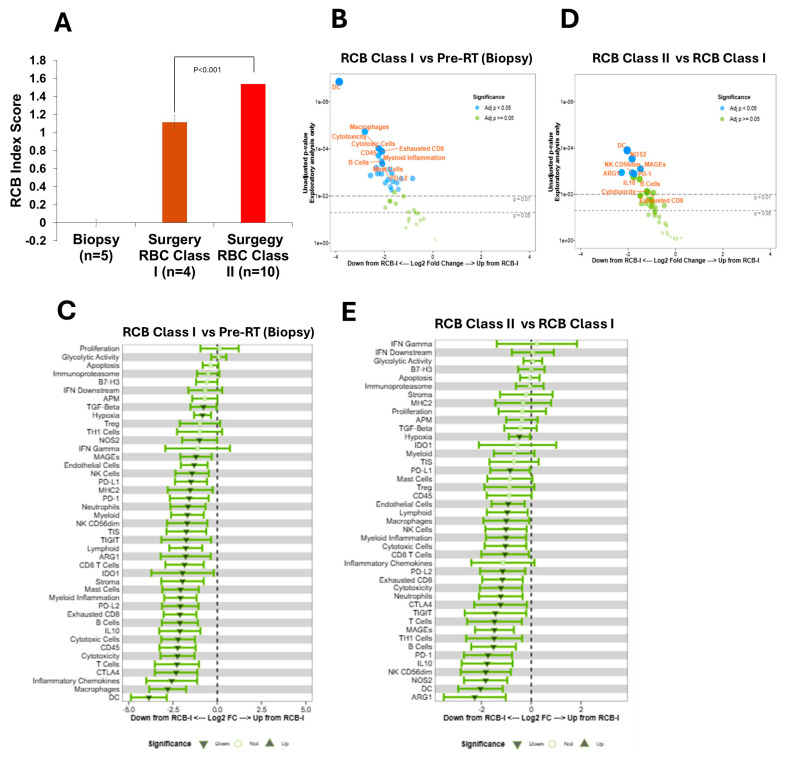
**RT-induced immunosuppressive TME with increasing RCB class in BC tumors**. (**A**) Analysis of RCB index score in biopsy and surgery (RCB class I and II) patients’ tumors. *p*  <  0.01 compared to RCB class I tumors (*n* = 4). (**B**,**C**) The volcanic and forest graph (IO360) represents the impact of RT on key immune cell markers and pathway regulation in biopsy vs. RCB class I tumors. (**D**,**E**) The volcanic and forest graph represents the impact of RT on key immune cell markers and pathway regulation in RCB class I vs. RCB class II tumors.

RCB is a standardized pathology scoring system that quantifies the amount of remaining tumor cells in the breast and lymph nodes after neoadjuvant therapy [26]. In our cohort study, we analyze the RBC class and score index tumors (MD Anderson Cancer Center, Houston, RBC score) for biopsy and post-RT. Our data clearly demonstrates that biopsy samples (*n* = 5) do not show the RCB class or score index; however, out of 15 post-RT tumor samples, one tumor sample (SA-20-6185) shows a pathologic complete response (pCR; the absence of invasive cancer in the breast), four tumors show RCB class I, and ten tumors show RCB class II (Table S1 and Figure 7A). Comparative analysis suggests that after RT treatment, RCB class II tumors show a significantly higher RCB index score compared with RCB class I BC tumors (*p* < 0.001), suggesting that RT enhances RCB in BC tumors.

Based on overall gene expression pattern (IO360) and as presented in the volcanic plot, our data suggest that compared to the baseline/biopsy tumor, RT significantly reduced the overall population of NK cytotoxic cells, B cells, and CD45 T and B cells and exhausted CD8T cells and PD-L2-expressing immune cells such as dendritic cells, T cells (specifically CD4+ cells, and CD8+ cells) and macrophages in RCB class I tumors (Figure 7B,C). Similarly, dendritic cells (DC), NK *CD56dm* cells, expression of immune cell *PD-1*, *NOS2*, and tumorigenic MAGE-related gene expression are downregulated in RBC class II BC tumors compared with RBC class I after RT treatment (Figure 7D,E). Note that Figure 7E is adapted from a previously published review article [16] and used in the current manuscript for comparative analysis. As indicated in Figure 7C,E, we comparatively presented the overall downregulation of immune priming between biopsy to RCB I to RCB II BC tumors after RT. The data clearly suggest that RT overall suppressed immune priming in BC tumors and increased cytotoxicity and RCB, which may cause radioresistance or therapy resistance in BC.

## 3. Discussion

**Figure 8:**
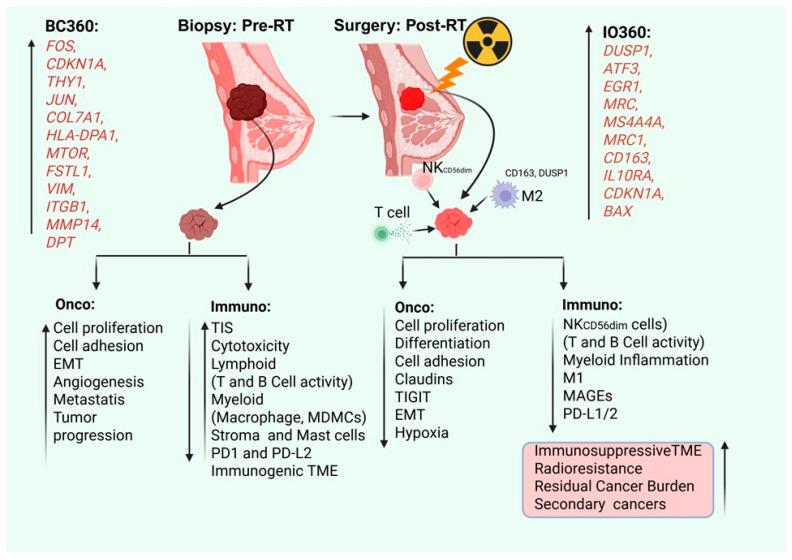
A schematic illustrates the effect of RT on the expression of key genes involved in oncogenic and immunogenic signaling. RT impacts tumor cell processes (phenotype) and modulates immune cell activity where presented. RT’s role in creating an immunosuppressive TME with increasing RCB class in secondary BC tumors was emphasized. The illustration was created in BioRender. Seneviratne, D. (2026) https://BioRender.com/pk5pz93 accessed on 5 October 2025.

As depicted in Figure 8, the analysis of gene biomarker expression and evaluation of associated pathways can provide a clearer picture of the therapeutic potential of RT, as well as its associated side effects and therapy resistance parameters. This study will provide valuable gene information for the consideration of other targeted therapies, such as chemotherapy and immunotherapy, for BC treatments. BC360 profiling showed several genes are predominantly associated with pro-tumorigenic functions such as tissue repair, survival, invasion, metabolic adaptation, and apoptosis. For example, *FOS* acts as a pro-oncogene; in combination with c-JUN, it forms the activator protein-1 (AP-1) transcription factor complex, which facilitates the expression of genes involved in cell proliferation, differentiation, and transformation [27]. Activation of *MTOR* following RT enables DNA damage recovery and improves survival and metabolic adaptation in tumor cells [28]. Previous studies have reported that targeting mTOR with inhibitors such as rapamycin, everolimus, or temsirolimus has been shown to sensitize breast cancer cells to radiation, both in vitro and in clinical settings [29,30,31,32]. Similarly, *JUN*, a key component of the *AP-1* transcription complex, controls transcription of genes involved in survival, proliferation, and stress-response, contributing to adaptive radioresistance [32]. ITGB1 (integrin β1) contributes to cell adhesion-mediated radioresistance (CAM-RR) and promotes invasion and metastasis post-RT in many cancers [33,34], and preclinical models of breast cancer have also indicated that blockade of integrin β1 enhances radiosensitivity [35]. Upregulation of *MMP14* has been reported to promote extracellular matrix remodeling, invasion, and angiogenesis, facilitating tumor progression following irradiation [36]. Vimentin (*VIM*), a classical EMT marker, reflects pro-metastatic reprogramming, with EMT-associated pathways contributing to radioresistance and invasion [37]. Among these, *HLA-DPA1* (a component of MHC-II expressed by tumor cells) is known to activate adaptive immune responses. The higher expression of stromal cell gene markers *THY1* (*CD90*), *FSTL1* (follistatin-like 1), and *HIF1A* suggests enhanced fibroblast activation, stromal remodeling, and fibrosis, potentially creating a tumor-supportive and hypoxic microenvironment that may impair the efficacy of RT [38,39].

On the other hand, the IO360 immune panel identified an upregulation of *CDKN1A*, *DUSP1*, *EGR1*, *BAX*, *ATF3*, *LIF*, *CD163*, MRC1, and several other genes. *CDKN1A* (p21) is a tumor suppressor protein that regulates the cell cycle and is involved in DNA repair. The tumor suppressor p53 regulates p21, which inhibits cyclin-dependent kinase (CDK) complexes, thus regulating cell apoptosis, senescence, and DNA damage response [40]. Upregulation of *DUSP1* (dual-specificity protein phosphatase) regulates context-dependent roles in cancer progression or suppression, and aberrant *DUSP1* regulation in cancer cells causes resistance to chemotherapy and radiotherapy and also facilitates immune evasion within the BC-TME and triple-negative BC (TNBC) [41,42]. Upregulation of the stress-responsive transcription factor that supports DNA repair, the *ATF3* gene, modulates macrophage polarization in tumors [43], indicating that RT induces DNA damage response, as expected, and creates a unique TME in BC tumors. Additionally, our data suggest that gene markers of M2-like TAMs, including *CD163*, *MRC1* (*CD206*), and *MS4A4A* expression, were high (enriched) in post-RT-treated BC tumors, consistent with an anti-inflammatory and pro-tumoral immune phenotype. Expression of these biomarkers was associated with poor prognosis and therapy resistance [44,45]. Upregulation of macrophage M2-associated *CD163* and *IL10RA* indicates enhanced IL-10-mediated immunosuppression, increasing anti-inflammatory signaling and favoring immune evasion. Similarly, increased *CCR2* expression suggests recruitment of circulating monocytes that differentiate into immunosuppressive TAMs, establishing a negative feedback loop that limits the anti-tumor effects of RT [46,47]. These findings highlight the identification of actionable targets for improving radiosensitivity and anti-tumor immunity. Tumor-intrinsic signaling pathways, including factors such as p21, mTOR, JUN/AP-1, ITGB1, DUSP1, and MMP14, represent potential candidates for radiosensitization. TAM recruitment and polarization can be mitigated via the use of CCR2 antagonists, CD163/CD206-targeting antibodies, or MS4A4A blockade, potentially shifting the TME toward a pro-inflammatory milieu. IL-10/IL-10R blockade may further enhance T cell-mediated anti-tumor responses [47]. Additionally, the preservation of beneficial immune-modulatory changes, such as *HLA-DPA1* upregulation, may synergize with immune checkpoint blockade (anti-PD-1/PD-L1 or anti-CTLA-4) to enhance adaptive immunity [48,49]. Targeting myeloid cells, such as macrophages and monocytic myeloid-derived suppressor cells (M-MDSCs), may improve immunogenic TME. Inhibition of MDSCs using an arginase-1 inhibitor (CB1158) or phosphodiesterase-5 inhibitor (tadalafil) decreased RT-mediated induction of lymphopenia, which could increase patient survival, as shown in patients with glioblastoma (GBM) and in preclinical mouse models [50,51].

Since RT induced TIS, as indicated by BC360 and IO360 panels, this inflammatory signaling may work in two ways: (1) increased cytotoxicity suppresses cell proliferation and adhesion and promotes apoptosis, (2) TIS may modulate mammary stemness and attract stromal, endothelial, and immune cells into the TME and may cause redioresistance. Our data suggests that RT induces mammary stemness in BC tumors by upregulating *VIM*, *TCF4*, *VCAN*, *SNAI2*, and *FSTL1* stemness genes. RT-mediated intrinsic cancer stem cell (CSC) generation has already been reported [52,53,54], which promotes radioresistance and increases the risk of tumor recurrence. Blocking of NF-kβ pathway by disulfiram (DSF) and Copper (Cu2+) significantly inhibited mammary primary tumor growth (79.4%) and lung metastasis in mice [53], suggesting that inhibition/targeting of stemness gene expression may increase radiosensitivity in BC tumors. RT increased the population of tumor-associated stromal cells, such as fibroblasts (CAFs), endothelial cells, and myeloid cells. The accumulation of these cells contributes to creating a unique permissive TME that can promote tumor progression, invasion, and treatment resistance [55]. RT increased stromal cells, endothelial cells, and mast cells in BC TEM after post-RT. RT also modulated B and T cell activity, as reported [56]. Our data also suggests that a significantly high score of B and T cells (CD45 and CD8 T) was observed in the BC-TME. Significant upregulation of macrophage-associated *CD163* and *DUSP1* and tumor-associated *DUSP1* and other genes may create an anti-inflammatory BC-TME, which leads to increased RCB in the BC tumor. Moreover, a decreased overall expression of genes associated with NK cells, NK*CD56dm*, CD45 T and B cells, DC cells, *PD-1* (immune cell), NOS2, and tumorigenic MAGEs in RBC class II BC tumors was observed compared with RBC class I tumors after RT treatment, clearly indicating that RT creates an immunosuppressive TME. The important role and significance of TAM-M2 *CD163* and *DUSP1*, which are induced post-RT, are currently under investigation in our laboratory.

While our findings highlight the dual impact of RT on oncogenic and immunogenic pathways in BC, several limitations should be considered. The relatively small sample size and limited number of pre-radiotherapy biopsy samples reduce statistical power and may introduce sampling bias. In addition, the largely unpaired study design limits the ability to attribute observed molecular changes to RT directly. The cohort is predominantly composed of Luminal A tumors, which may restrict the generalizability of these findings across more aggressive BC subtypes. Furthermore, the use of targeted NanoString panels confines the analysis to predefined gene sets, and the findings remain largely correlative rather than functional. Potential confounding clinical variables, including variability in treatment modalities and patient-specific characteristics, were not fully controlled for. Finally, the absence of long-term clinical outcomes limits the ability to determine the prognostic significance of these observations, warranting validation in larger prospective studies. On the other hand, this study gives direct evidence of RT’s impact on BC tumor oncogenic and immunogenic expression profiling. Currently, in our laboratory, we are validating the detailed role of the top oncogenic and immunogenic regulatory biomarkers in order to design targeted therapies for managing BC and aggressive BC treatment options.

## 4. Materials and Methods

### 4.1. Breast Cancer Tumor Collection

The study was conducted under the title “A Pilot/Phase II Trial of Hypofractionated Radiotherapy to the Whole Breast Alone before Breast Conserving Surgery”. The study was approved by the ethics committee and the Institutional Review Board (IRB), protocol code 17-004130 and IRB modification # Mod17-004130-59, on 24 December 2024. All patients signed a consent form permitting the use of donated BC tissue. Between 2019 and 2020, patients with cT0-T2, N0, M0 BC were enrolled and treated with hypofractionated whole-breast RT, five fractions (5 Gy each day, for 5 days, in one week; total 25 Gy), as described earlier [57]. RT was delivered with proton beam therapy (PBT) or photons, and no RT boost or regional nodal irradiation was allowed. After 4–8 weeks, breast conservation surgery (BCS) was performed. In this study, we analyzed RT’s impact on 20 patients. We isolated 20 tumor tissues from 20 breast cancer patients, of which five tumor biopsy specimens were collected during diagnosis. These we call “Pre-RT tumor samples” or “biopsy” samples. After completion of RT and after 4–8 weeks, BCS was performed, and post-RT or surgical BC tumor samples were collected (Figure 1A and Table S1). One patient (ID: SF-18-3514) did not undergo surgery; however, four biopsy patients underwent RT. Detailed characteristics of patients and the study cohort are described in Supplementary Table S1. Therefore, in this cohort study, we used 5 biopsy samples (pre-RT) and 14 or 15 surgical samples (post-RT) for further analysis.

### 4.2. Quality Assessment of Clinical FFPE BC Tissue Specimens

Biopsy samples and post-RT surgical BC tumor specimens were collected as per approved clinical protocols. Pre-RT (biopsy) and post-RT (surgical) tumor tissue specimens were fixed with formalin and embedded in paraffin (FFPE) as described earlier [58]. FFPE block sections were cut at 5 µm and mounted onto positively charged slides. One section from each tissue sample was stained with hematoxylin and eosin (H and E), and the sample quality assessment was determined.

### 4.3. BC Tumor RNA Extraction and Characterization

Total RNAs from pre-RT and post-RT samples were isolated using the RNeasy FFPE Kit (Qiagen Cat #73504 Germantown, MD, USA) as per the manufacturer’s instructions. The RNA quality and concentrations were estimated using the NanoDrop ND-1000 spectrophotometer (Wilmington, DE, USA, and RNA integrity analysis was performed by using the Agilent RNA 6000 Nano Kit and Agilent 2100 Bioanalyzer (Santa Clara, CA, USA) Samples were sent to NanoString (Bothell, WA, USA) for further gene expression analysis.

### 4.4. RNA Sequencing and Data Analysis

For RNA sequencing, the NanoString (nCounter Run) technology was used, and we applied two approaches to analyze and validate sequencing data. We used the nCounter BC-specific 360 panel (BC360) and immune-oncology 360 (IO360) panCancer panels for analyzing RT’s effect on BC-specific onco-markers and immuno-oncogenic markers (gene expression) analysis. For BC360 panel sequencing, out of 20, 19 BC samples passed all quality control (QC) tests, which include 5 pre-RT (biopsy) and 14 post-RT (surgery), and in IO360, five pre-RT (biopsy) and 15 post-RT (surgery) samples passed quality control (QC) tests (Figure 1A). The samples with failed housekeeping geomean QC were excluded from the analysis. Total RNA (50 ng in 5 μL) was incubated at 65 °C for 12–16 h with a 3′ biotinylated capture probe and a 5′ reporter probe tagged with a fluorescent barcode from the custom (BC360 or IO360) gene expression code set. After hybridization, the sample mixtures were run on the NanoString nCounter preparation station, and excess biotinylated capture probe and reporter probe were removed. A streptavidin-coated cartridge was used to immobilize transcript-specific ternary complexes. Finally, samples were scanned on the nCounter Digital Analyzer with maximum resolution.

### 4.5. Gene Expression Normalization

Normalization in BC360 differs from normalization in nSolver. The goal is to adjust cartridge differences using either a panel standard or reference sample (*BC360_PS1* and *BC360_PS2* for BC360 *or PanelSTD* for IO360) such that comparisons can be made between the scores across batches. The panel standard is a DNA oligo blend containing all BC360 probe target sequences, which is run on each cartridge within the experiment for normalization of non-PAM50 genes (PAM50: a 50-gene signature used in BC tumor classification: Luminal A, Luminal B, HER2-enriched, Basal-like, and Normal-like); while the reference sample is an RNA oligo blend containing PAM50 probe target sequences for PAM50 genes. Normalization takes place in two steps. The first step differs depending on whether the genes are in PAM50 or TISs (tumor inflammation signatures) or not. Zero counts on the raw scale are converted to ones before normalization. Gene normalization of PAM-50 non-PAM50, TIS, and non-TIS was performed using a ratio of expression value to the geometric mean of all respective panels and signatures. Genes in the PAM50 signature are additionally normalized using a ratio of the housekeeper-normalized data and a reference sample run on the same code set lot from a NanoString archive. Final adjustments of gene expression normalization were carried out using the housekeeper-normalized and panel standard-normalized data, which were Log (2)-transformed as described in the manufacturer’s protocol.

### 4.6. Differential Expression Analysis

Differential expressions are fit on a per-gene or per-signature basis using a linear model for analyses without a blocking factor. The statistical model uses the expression value or signature score as the dependent variable and fits a grouping variable as a fixed effect to test for differences in the levels of that grouping variable. Expression (gene or signature) = μ + Group + ε. The statistical significance and magnitude of gene expression (fold change) were determined by adjusting *p*-values within each analysis, gene, or signature, and at the grouping variable level, using a *t*-test followed by the Benjamini and Yekutieli False Discovery Rate (FDR) adjustment to account for correlations among the tests. In addition, gene expression differences between biopsy and surgery groups were analyzed using a two-tailed Student’s *t*-test (unpaired). A *p*-value of <0.05 was considered statistically significant. GraphPad Prism 9 software (La Jolla, CA, USA) was used for analysis, comparative gene expression, score analysis of each pathway, and graph plotting. The RNA sequencing data and associated protocols of BC360 and IO360 panels were deposited in the GEO database with accession number GSE307387.

## 5. Conclusions

In this report, we analyzed the direct effect of RT on the BC tumor and the regulation of oncogenic and immunogenic profiling in a patient with BC in vivo. Our study highlights the dual nature of RT-induced transcriptional and immunological changes in BC, and we identify several key biomarkers that are sensitized to RT or radioresistance (Figure 8). RT enhances systemic responses for immunotherapy and must be used in adjuvant, neoadjuvant, and palliative treatments and in managing secondary BC. Radiation-mediated induction of these gene biomarkers and their significance in BC treatments are under investigation in our laboratory currently. Collectively, these findings highlight the impact of RT on oncogenic and immunogenic profiling in BC and support biomarker-guided strategies to optimize RT and combination therapies. Prospective validation and integration with clinical endpoints will be essential for translation into precision oncology.

## Data Availability

The original contributions presented in this study are included in the article/Supplementary Material. Further inquiries can be directed to the corresponding authors.

## Supplementary Materials

The following supporting information can be downloaded at: https://www.mdpi.com/article/10.3390/ijms27073227/s1.

## Abbreviations

BC	Breast Cancer
TNBC	Triple-Negative Breast Cancer
RT	Radiation Therapy
pCR	Pathological Complete Response
RCB	Residual Cancer Burden
TME	Tumor Microenvironment
EMT	Epithelial–Mesenchymal Transition
CSC	Cancer Stem Cell
PD-1	Programmed Cell Death Protein 1
PD-L1	Programmed Death-Ligand 1
CTLA4	Cytotoxic T-Lymphocyte-Associated Protein 4
CD3	T Cell Receptor Complex
CD4^+^	Helper T Cell
CD8^+^	Cytotoxic T Cell
CD45	Leukocyte Common Antigen
CD79A	B Cell Marker
CD163	M2 Macrophage Marker
MRC1/CD206	M2-Like Macrophage Marker
Treg	Regulatory T Cell
NK	Natural Killer (Cells)
PRF1	Perforin
GZMB	Granzyme B
GZMA	Granzyme A
CCL2	Chemokine (C-C motif) Ligand 2
CXCL9	Chemokine (C-X-C motif) Ligand 9
CXCL10	Chemokine (C-X-C motif) Ligand 10
IL6	Interleukin-6
IFN-γ	Interferon Gamma
STAT1	Signal Transducer and Activator of Transcription 1
mTOR	Mechanistic Target of Rapamycin
PTEN	Phosphatase and Tensin Homolog
FOxA1	Forkhead Box A1
ER/ESR1	Estrogen Receptor
PR/PGR	Progesterone Receptor
HER2	Human Epidermal Growth Factor Receptor 2
AR	Androgen Receptor
BCL2	B Cell Lymphoma 2
BAX	BCL-2-Associated X Protein
TGF-β	Transforming Growth Factor Beta
FAP	Fibroblast Activation Protein
COL6A3	Collagen Type VI Alpha 3 Chain
CDH5	VE-Cadherin (Endothelial Marker)
VIM	Vimentin
SNAI2	Snail Family Transcriptional Repressor 2
SOX2	SRY-Box Transcription Factor 2
TCF4	Transcription Factor 4
TIS	Tumor-Inflammation Signature
DEG	Differentially Expressed Gene
FDR	False Discovery Rate
IO360	Immuno-Oncology 360 NanoString Panel
TILs	Tumor-Infiltrating Lymphocytes
