# Supplementary material for: Characterization of Oncogenic and Immunogenic Profiling in Patients with Breast Cancer Tumors After Radiation Therapy
Source: Int J Mol Sci. 2026 Apr 2;27(7):3227. doi: 10.3390/ijms27073227 (PMC13073414; doi:10.3390/ijms27073227)

Fig. S1

BC360: BC-specific oncology panel

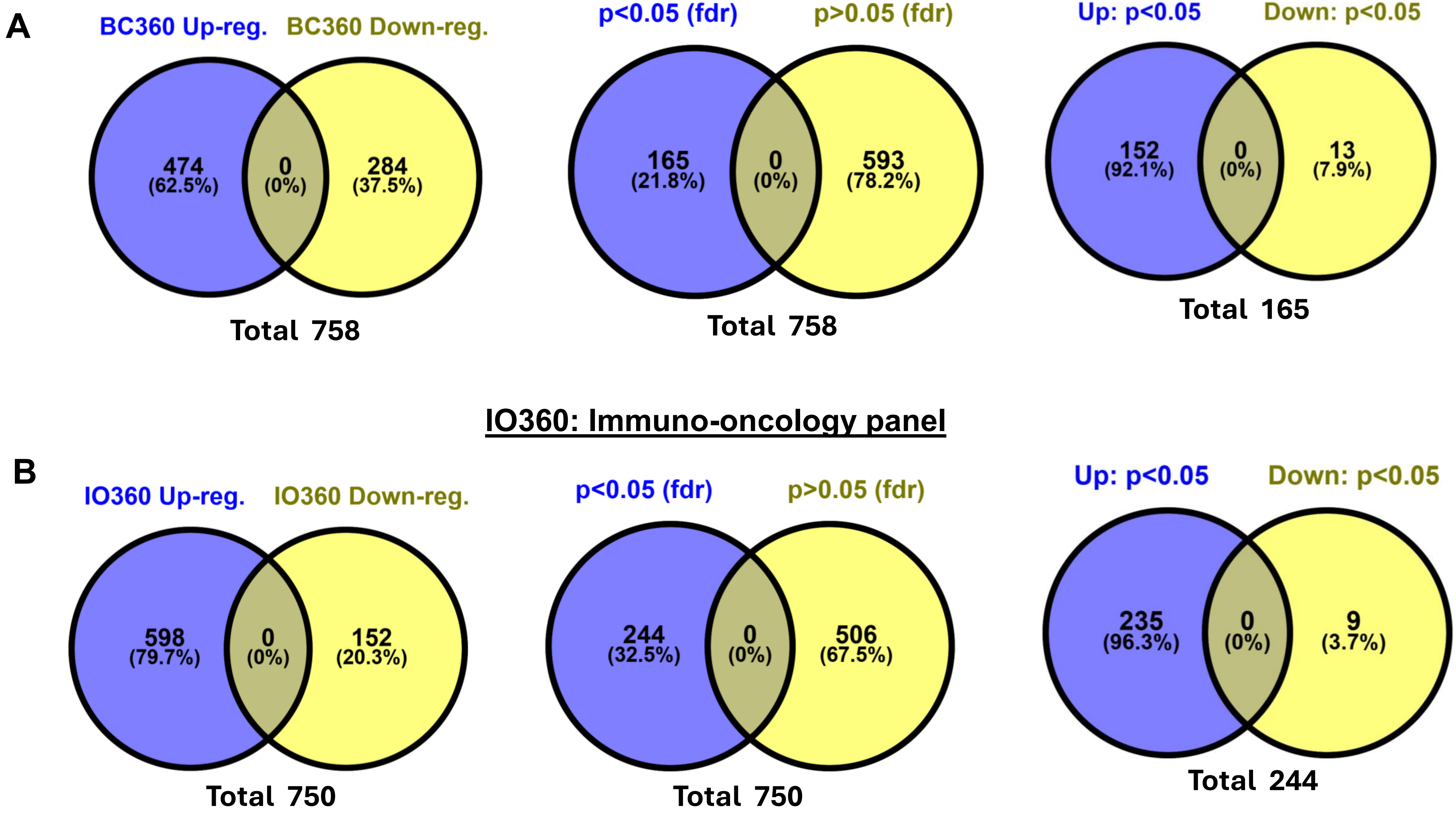

Supplement: Supplementary file 1 [file ijms-27-03227-s001.zip › S1.pdf]
