# Supplementary material for: Characterization of Oncogenic and Immunogenic Profiling in Patients with Breast Cancer Tumors After Radiation Therapy
Source: Int J Mol Sci. 2026 Apr 2;27(7):3227. doi: 10.3390/ijms27073227 (PMC13073414; doi:10.3390/ijms27073227)
Supplement: Supplementary file 1 [file ijms-27-03227-s001.zip › Supplementary table and figure legends.pdf]

International Journal of
Molecular Sciences

MDPI

Supplementary table and figure legends

Table S1: The table represents the identification of patients with BC and BC tumor characterization.

Figure S1: RT modulates Gene expression in BC tumors. A) A Venn diagram represents up- and downregulated (left panel), significantly and not significantly (total) up- and downregulated (middle panel), and only significantly up- and downregulated gene numbers (right panel) and gene percentages from BC360 nCounter analysis. **B**) A Venn diagram represents the total up- and downregulated (left panel), total significantly and not significantly up- and downregulated (middle panel), and only the significantly up- and downregulated gene numbers and gene percentages (right panel) from IO360 nCounter analysis. The statistical significance was determined by analyzing *p*-value adjustment using a t-test followed by the Benjamini and Yekutieli False Discovery Rate (FDR) adjustment.

Figure S2: Identification of DGEs in BC tumors after post-RT. A) Upper panel: From BC360 and IO360 panels, the genes showed significantly altered expression BC360-specific, IO-360 specific, and common between BC-360 and IO360 panels were analyzed and plotted using Venny 2.1.0 bioinformatic tool (https://bioinfogp.cnb.csic.es/tools/venny/; access date September 25, 2025). Lower panel: The expression and distribution of BC360 panel-specific (165) upregulated, downregulated, and common genes ($p < 0.05$), as well as IO-360 (244) panel-specific upregulated and downregulated, and common genes ($p < 0.05$), are presented using Venny 2.1.0. **B &C)** Heat maps represent the top 31 genes upregulated ($p<0.001$) from BC360 and IO360 analysis and after post-RT in BC tumors (upper panels). Heat maps represent significantly downregulated genes ($p<0.05$) from BC360 and IO360 analysis after post-RT in BC tumors compared to biopsy samples (lower panels).

Figure S3: RT decreased BRCAness and genomic risk in BC tumors. A) RT effect of regulation of the BRCAness-associated gene expression in patients (heat map) and biopsy (n=5) vs surgery (n=14) (BRCAness-associated) gene expression and BRCAness score were presented with significance. **B)** RT impact on regulation of the HRD-associated gene expression in patients (heat map) and biopsy vs surgery HRD-associated gene expression and HRD score were presented. **C**) RT impact on overall genomic risk (heat map) and biopsy vs surgery genomic risk score was analyzed based on gene expression and plotted. **D**) The comparative effect of RT on the genomic risk score in BC tumors from four patients (biopsy vs surgery; individual and combined; upper and lower panels) was calculated and plotted. *$P < 0.05$, **$P < 0.01$, and ***$P < 0.01$ related to biopsy tumor samples.

Figure S4: Effect of RT on ER and p53 signaling gene expression. A &B) BC360 analysis: Heat maps represent the effect of RT on ER and p53 signaling gene expression in biopsy (*n*=5) and surgery (*n*=14) tumor samples (upper panel). The relative *ESR1*, *HER2* gene score, and ER and p53 signaling scores were calculated from pre-RT and post-RT and plotted (lower panels) with significance.

Figure S5: Effect of RT on key gene biomarker score. A -C) BC360 analysis: RT impact on regulation of *AR*, *RB1*, *PGR*, *PTEN*, *FOXA1*, *SOX2*, *TGF-β*, *INF-γ*, and hypoxia score (**A**), *CDK4*, *CDK6*, *B7-H3*, *IDO1*, *TIGT*, CD8 T cell, macrophages, and mast cell scores (**B**), and *PD-L1*, *PD-L2*, and T cell-specific *PD-1* gene scores (**C**) were presented (biopsy *n*=5 vs surgery *n*=14). *$P < 0.05$, **$P < 0.01$, and ***$P < 0.01$ related to biopsy tumor samples.

Figure S6: RT affects MHCII and APM-related gene regulation. A) The effect of RT on MHC II-associated overall gene expression in BC patients (heat map), overall MHC II-associated overall gene score, and key genes' fold change were presented. **B**) The effect of RT on APM-associated overall gene expression in BC patients (heat map), overall APM-associated gene score, and key genes associated with APM-regulated (fold change) were presented. *$P < 0.05$ related to biopsy tumor samples.

Figure S7: RT sensitized BC tumors and modulated residual cancer burden. A) BC360 analysis of the volcanic plot represented key upregulated genes (*FOS*, *CDKN1A*, *JUN*, *GDF5*, *BAX*, *FGL7/2*, and *DSC2*) after RT ($p<0.01$). **B**) The forest graph represents the impact of RT on key genes and pathways (upregulated and downregulated associated with

BC tumors (biopsy vs surgery). Arrows indicated the significant pathways affected after RT. **C**) Effect of RT on RCB and associated gene expression and pathways regulation were presented as volcano plots [Upper panel: RCB class I vs biopsy class; Lower panel: RCB class I vs RCB class II. **D&E**) Classification of BC tumor subtype with aggressiveness used in this cohort study was presented (**D**). A sunburst bar plot displays biological/clinical features around a circle, with bar lengths representing values or scores of Basal, LumB, and LumA BC subtype (as shown in the center) (**E**). LumB and LumA, the same tumor samples were presented (biopsy vs surgery), and only the surgery (RT+) basal tumor sample was included. Note: Basal-type biopsy samples are lacking in our cohort study.

Figure S8: RT induced TIS in BC tumor. A and B) IO360 gene expression profiling induced TIS-associated gene expression and overall TIS score in BC tumor after post-RT (left and right panels). *$P < 0.05$, and **$P < 0.01$ related to biopsy tumor samples (n=5).

Figure S9: RT regulates cell proliferation, cell cytotoxicity, and apoptosis gene signature in BC tumors. A) IO360 analysis: Effect of RT on tumor cell proliferation-related gene expression was present as a heat map, fold change, and overall proliferation score. *p-values* are presented within graphs. **B**) Heat map analysis, DGEs analysis, and NK cell gene analysis score represent RT enhanced cell cytotoxicity in BC tumors (all panels). **C**) RT induced tumor cell apoptosis in BC tumors. Left panel: heatmap of apoptotic gene expression in BC patients' tumors (biopsy n=5 and surgery n=15). Middle and left panels: DGEs and score of apoptotic gene expression between biopsy vs surgery tumor samples. *$P < 0.05$, **$P < 0.01$, and ***$P < 0.01$ (n=15) related to biopsy tumor samples (n=5).

Figure S10: Effect of RT on lymphoid gene panel expression. A) IO360 analysis: RT effect of lymphoid gene signature in biopsy and surgery tumor samples, and lymphoid gene score were presented. **B-F**) Relative RT effect on lymphoid-associated gene expression panels, such as CDs, STAT/JAK1, etc., granzymes, and chemokines (fold change) between biopsy (n=5) and surgery BC tumor samples (n=15) were presented with significance. *$P < 0.05$ and **$P < 0.01$ related to biopsy tumor samples.

Figure S11: Effect of RT on myeloid gene panel expression. A-D) IO360 analysis: RT effect of myeloid gene signature in overall and biopsy (n=5) and surgery (n=15) tumor samples (heat map) were presented (**A** and **B**). Relative RT effect on myeloid-associated gene expression panels as indicated (fold change) between biopsy vs surgery BC tumor samples, was presented with significance (**C**), and overall myeloid score (biopsy vs surgery) tumor samples (**D**) *$P < 0.05$ and **$P < 0.01$ related to biopsy tumor samples). *$P < 0.05$, **$P < 0.01$, and ***$P < 0.01$ related to biopsy tumor samples.

Figure S12: RT regulates MHC Class II (MHC II) gene expression in BC tumors. A) The heat map represents the effect of RT on DGEs associated with MHC II (left panel), MHC II-related gene expression (middle panel), and overall MHC II gene expression score (biopsy vs surgery) (right panel). **B**) RT effect on exhausted CD8 T cell score, and *CTLA4, PD-L1*, *PDL2*, *PD1*, and *ARG1* gene scores were calculated from the I0360 panel and plotted. **C**) RT effect on antigen processing machinery (APM) was analyzed, and associated with the heat maps, gene expression profile, and relative APM score and APM loss score in BC tumor were analyzed and plotted (upper and lower panels). *$P < 0.05$ and **$P < 0.01$ related to biopsy tumor samples (n=5).

Figure S13: RT regulated gene expression related to stroma cells, epithelial cells, and MAGEs in BC tumor. A) Impact of RT on stromal, endothelial, and MAGEs-related gene signature, score, and fold change of gene expression were calculated (IO360) and plotted as indicated. **B**) The overall and comparative mast cell-related score, B7-H3 (CD276), IDO1, and immunoproteasome scores were calculated (pre-RT vs post-RT) with significance. *$P < 0.05$ and **$P < 0.01$ related to biopsy tumor samples. *$P < 0.05$ and **$P < 0.01$related to biopsy tumor samples.
