# Supplementary material for: Characterization of Oncogenic and Immunogenic Profiling in Patients with Breast Cancer Tumors After Radiation Therapy
Source: Int J Mol Sci. 2026 Apr 2;27(7):3227. doi: 10.3390/ijms27073227 (PMC13073414; doi:10.3390/ijms27073227)
Supplement: Supplementary file 1 [file ijms-27-03227-s001.zip › Table S1.pdf]

Table S1

Sample ID	Time point	Race	Ethnicity	Age_RT	Breast	Multiple_lesions	Hist. type	Hist Grade	Stage	Staget	Stagem	Lymp-hodes	Tumor size	ER 0 = negative 1 = positive	PR 0 = negative 1 = positive	HER2 0= negative 1 = positive	# of Tertiary Lymphoid Structures (set 2)	TLS (0 vs 1 or more)	TILs (set 2)	TILs Status (10% cut-off)	MD Anderson RCB (set 2)	Residual Cancer Burden Class	Ki67 Scoring	Ki67 (1% cut-off)
SA-20-2900	biopsy	White	Not Hispanic or Latino	72	Left	No	DCIS	Grade I	0	T1a	M0	N0	0-2 cm	1	1	n/a								
SF-18-3513	biopsy	White	Not Hispanic or Latino	77	Left	Yes	Invasive ductal	Grade I	1A	T1a	M0	N0	0-2 cm	1	1	0								
SF-18-3514	biopsy	White	Not Hispanic or Latino	67	Right	No	Infiltrating ductal	Grade I	1A	T1a	M0	N0	0-2 cm	1	1	0								
SA-20-6775	biopsy	White	Not Hispanic or Latino	58	Left	No	Infiltrating ductal	Grade II	1A	T1a	M0	N0	0-2 cm	1	1	0								
SF-20-5585	biopsy	White	Not Hispanic or Latino	69	Right	No	Infiltrating ductal	Grade I	1B	T1a	M0	N0	0-2 cm	1	1	0								
FF-19-1774	surgery	White	Not Hispanic or Latino	77	Left	Yes	Invasive ductal	Grade I	1A	T1a	M0	N0	0-2 cm	1	1	0	0	0	<5%	Low	1.392	RCB-II	1-10%	High
FF-19-6658	surgery	White	Not Hispanic or Latino	61	Right	Yes	Infiltrating ductal	Grade I	1A	T1a	M0	N0	0-2 cm	1	1	0	13	1 or more	10%	High	1.592	RCB-II	1-10%	High
SA-19-17612	surgery	Asian	Not Hispanic or Latino	59	Right	No	Infiltrating lobular	Grade II	1A	T1a	M0	N0	0-2 cm	1	1	0	3	1 or more	<5%	Low	1.48	RCB-II	1-10%	High
SA-20-12711	surgery	White	Not Hispanic or Latino	58	Left	No	Infiltrating ductal	Grade II	1A	T1a	M0	N0	0-2 cm	1	1	0	5	1 or more	30%	High	1.934	RCB-II	51-60%	High
SA-20-14207	surgery	White	Not Hispanic or Latino	46	Right	No	Infiltrating ductal	Grade I	1A	T1a	M0	N0	0-2 cm	1	1	1	0	0	10%	High	1.128	RCB-I	<1%	Low
SA-20-2920	surgery	White	Hispanic or Latino	57	Left	No	Infiltrating ductal	Grade II	1A	T1a	M0	N0	0-2 cm	1	1	0	3	1 or more	5%	Low	1.369	RCB-II	1-10%	High
SA-20-6962	surgery	White	Not Hispanic or Latino	77	Left	No	Infiltrating ductal	Grade I	1B	T1a	M0	N0	0-2 cm	1	1	0	4	1 or more	10%	High	1.955	RCB-II	1-10%	High
SA-20-7073	surgery	White	Not Hispanic or Latino	44	Right	No	Infiltrating ductal	Grade II	1A	T1a	M0	N0	0-2 cm	1	1	0	0	0	10%	High	1.715	RCB-II	1-10%	High
SA-19-17482	surgery	White	Not Hispanic or Latino	57	Right	No	Infiltrating ductal	Grade I	1A	T1a	M0	N0	0-2 cm	1	1	0	0	0	5%	Low	1.1	RCB-I	1-10%	High
SA-19-18533	surgery	White	Not Hispanic or Latino	72	Left	No	Infiltrating lobular	Grade III	1A	T1a	M0	N0	0-2 cm	1	1	0	2	1 or more	5%	Low	1.389	RCB-II	<1%	Low
SA-20-12682	surgery	African American	Not Hispanic or Latino	67	Right	No	Infiltrating ductal	Grade I	1A	T1a	M0	N0	0-2 cm	1	1	n/a	1	1 or more	5%	Low	1.48	RCB-II	1-10%	High
SA-20-5757	surgery	White	Not Hispanic or Latino	70	Right	No	Infiltrating ductal										2	1 or more	<5%	Low	1.141	RCB-I		
SA-20-6175	surgery	White	Not Hispanic or Latino	60	Right	No	Infiltrating ductal	Grade II	1A	T1a	M0	N0	0-2 cm	1	1	n/a	0	0	30%	High	0.974	RCB-I	1-10%	High
SA-20-6185	surgery	White	Not Hispanic or Latino	72	Left	No	DCIS	Grade I	0	T1a	M0	N0	0-2 cm	1	1	n/a	n/a		n/a			pCR	n/a	
FF-20-5577	surgery	White	Not Hispanic or Latino	69	Right	No	Infiltrating ductal	Grade I	1B	T1a	M0	N0	0-2 cm	1	1	1	0	0	<5%	Low	1.841	RCB-II	1-10%	High
