# Supplementary figures and images for: Characterization of Oncogenic and Immunogenic Profiling in Patients with Breast Cancer Tumors After Radiation Therapy
Source: Int J Mol Sci. 2026 Apr 2;27(7):3227. doi: 10.3390/ijms27073227 (PMC13073414; doi:10.3390/ijms27073227)

**Fig. S10**

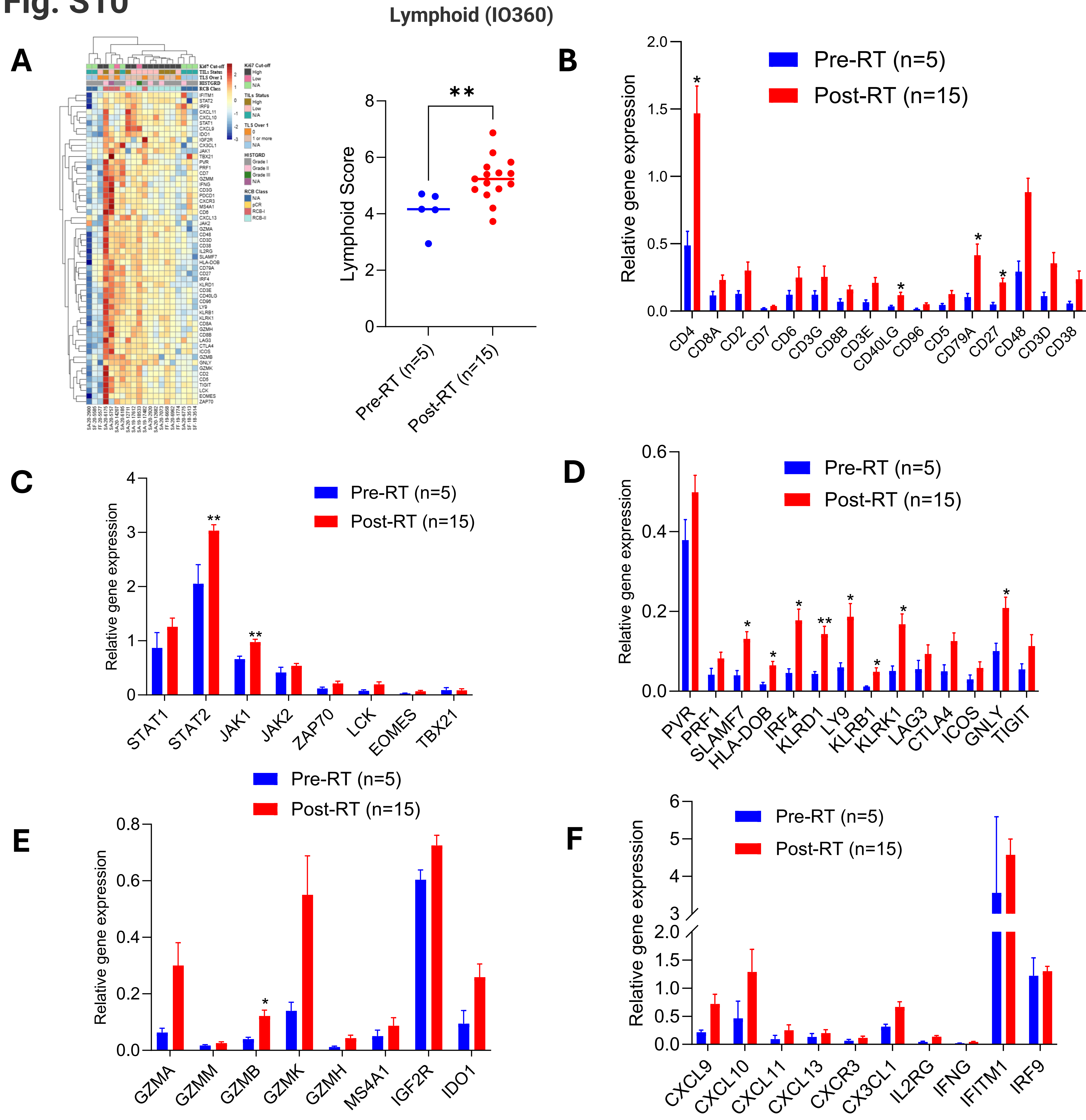

Supplement: Supplementary file 1 [file ijms-27-03227-s001.zip › S10.pdf]

**Fig. S11**

myeloid (IO360)

**A**

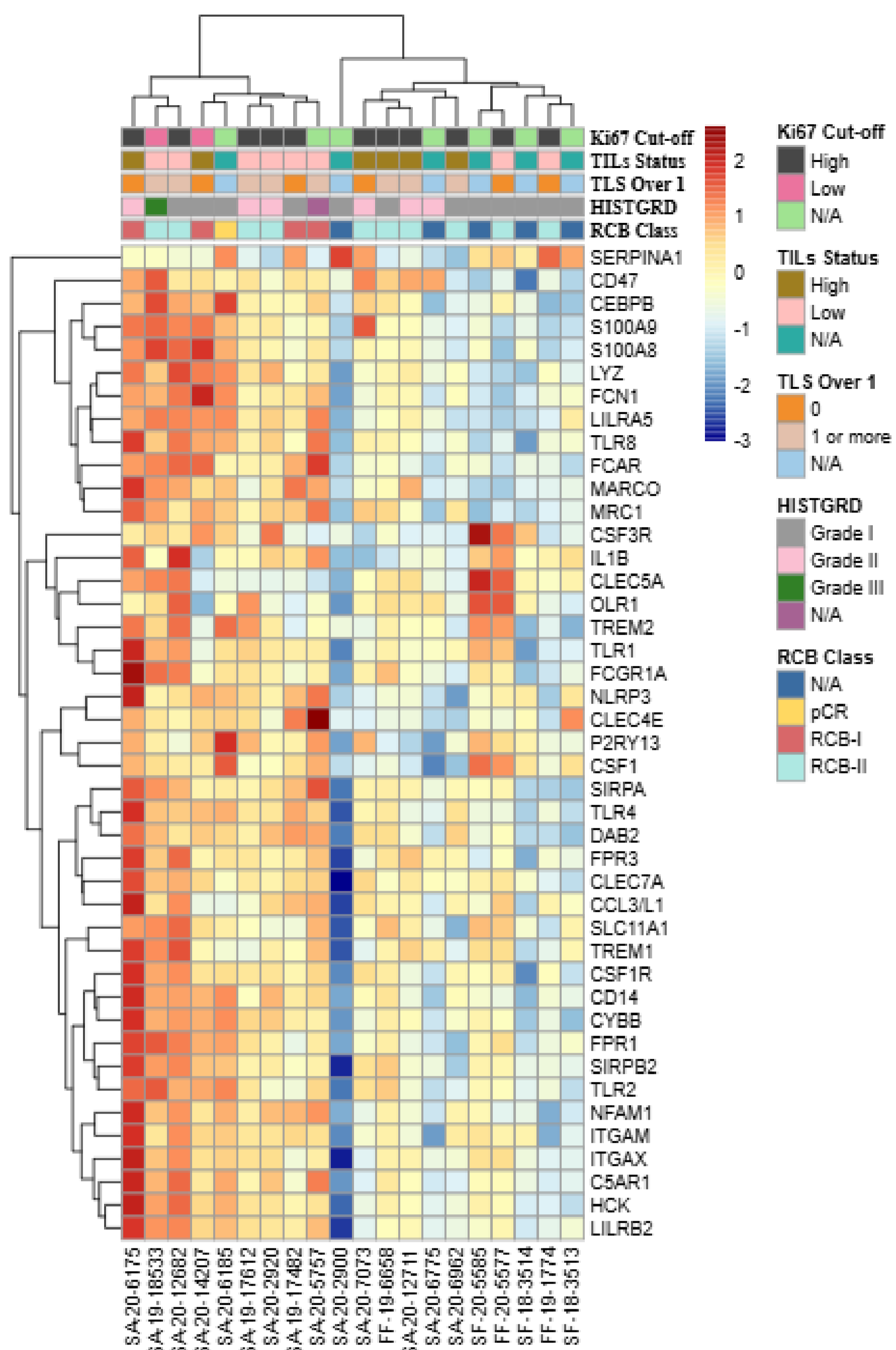

**B**

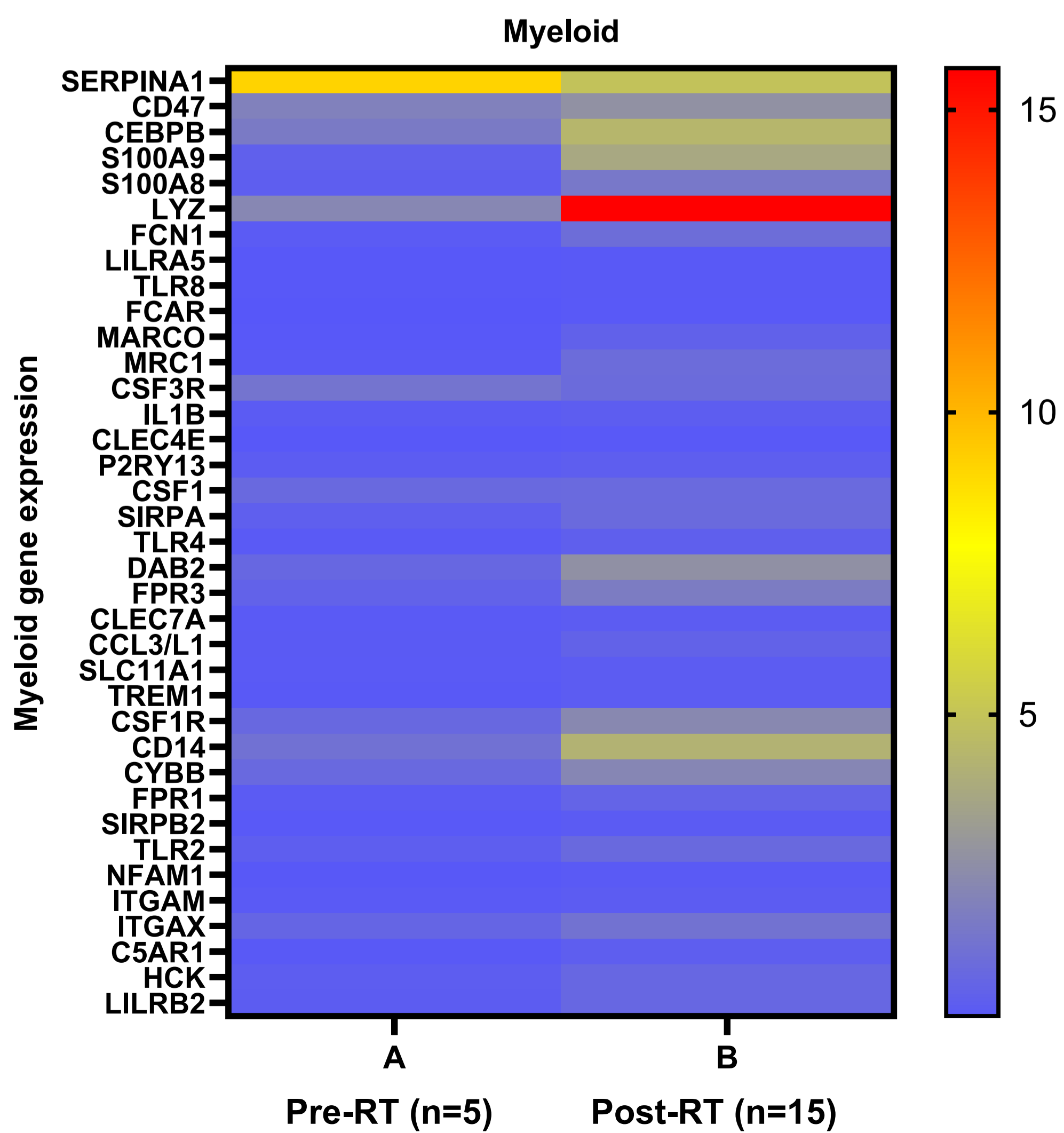

**C**

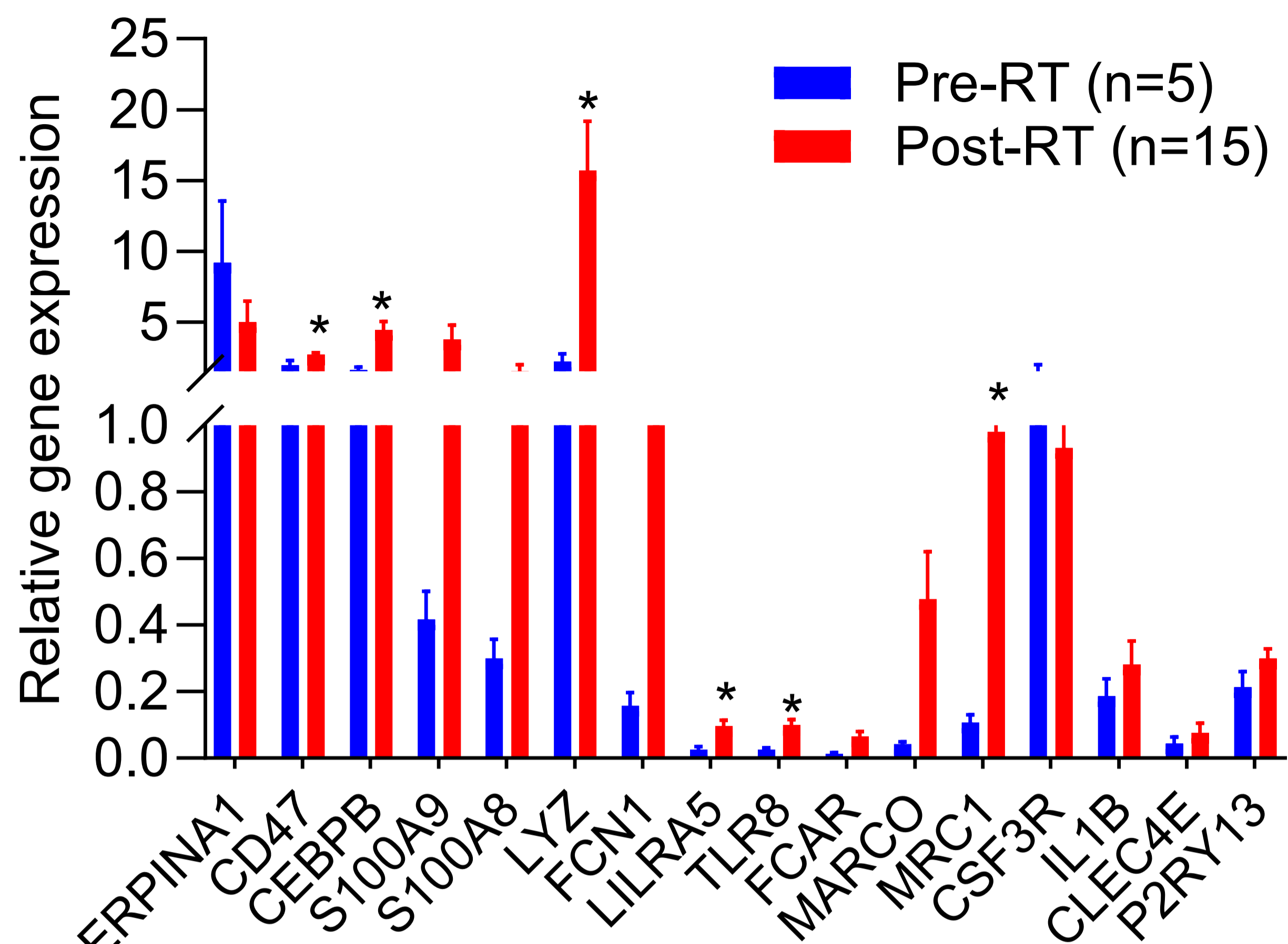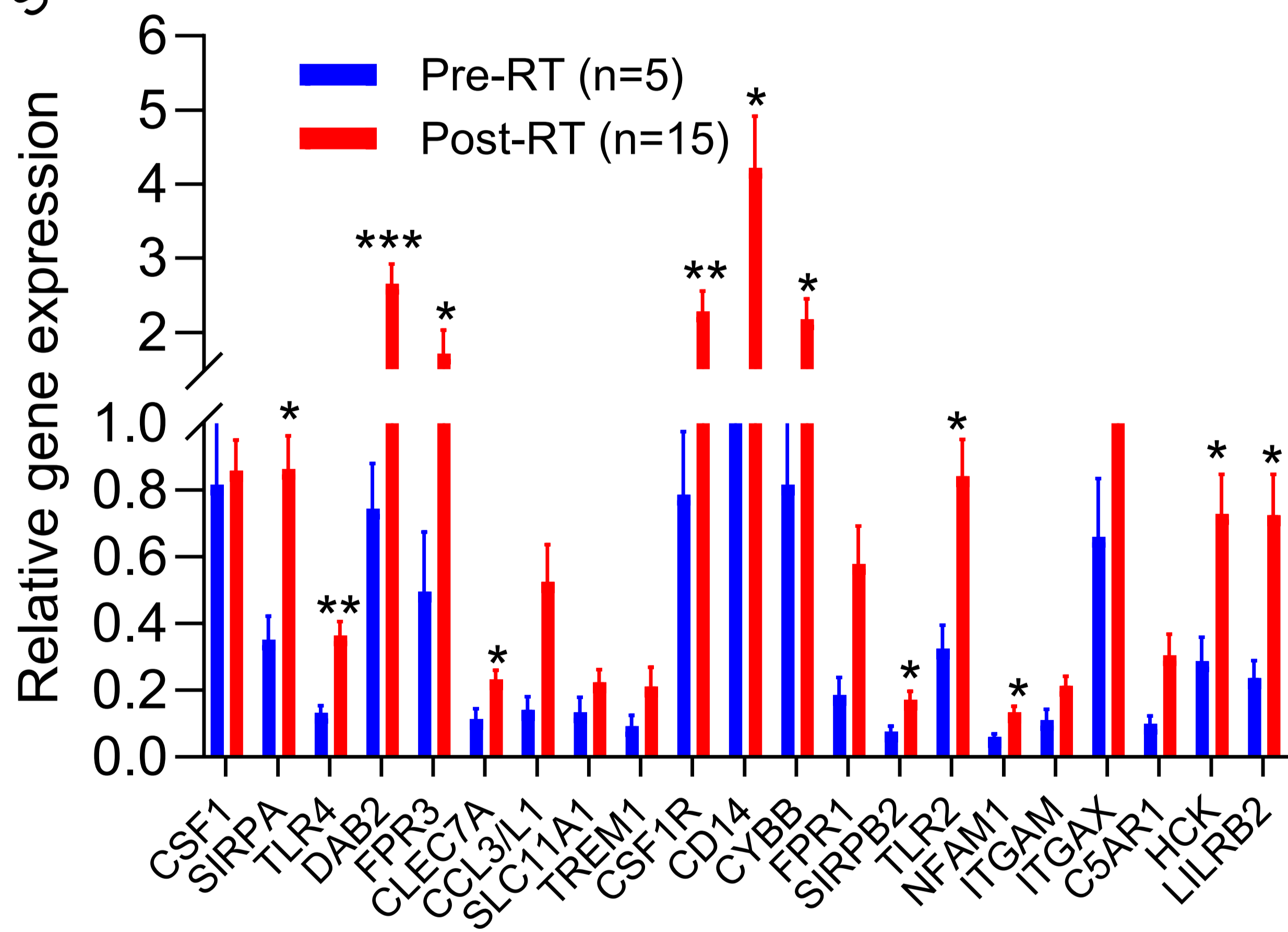

**D**

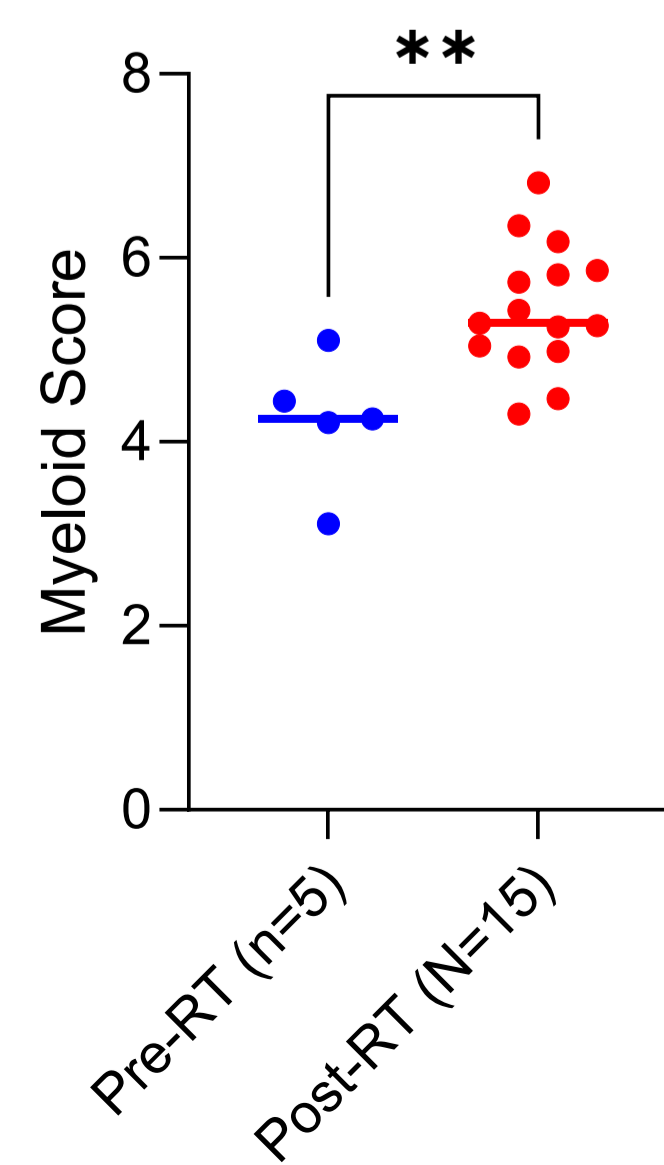

Supplement: Supplementary file 1 [file ijms-27-03227-s001.zip › S11.pdf]

**A**

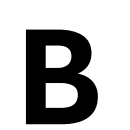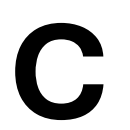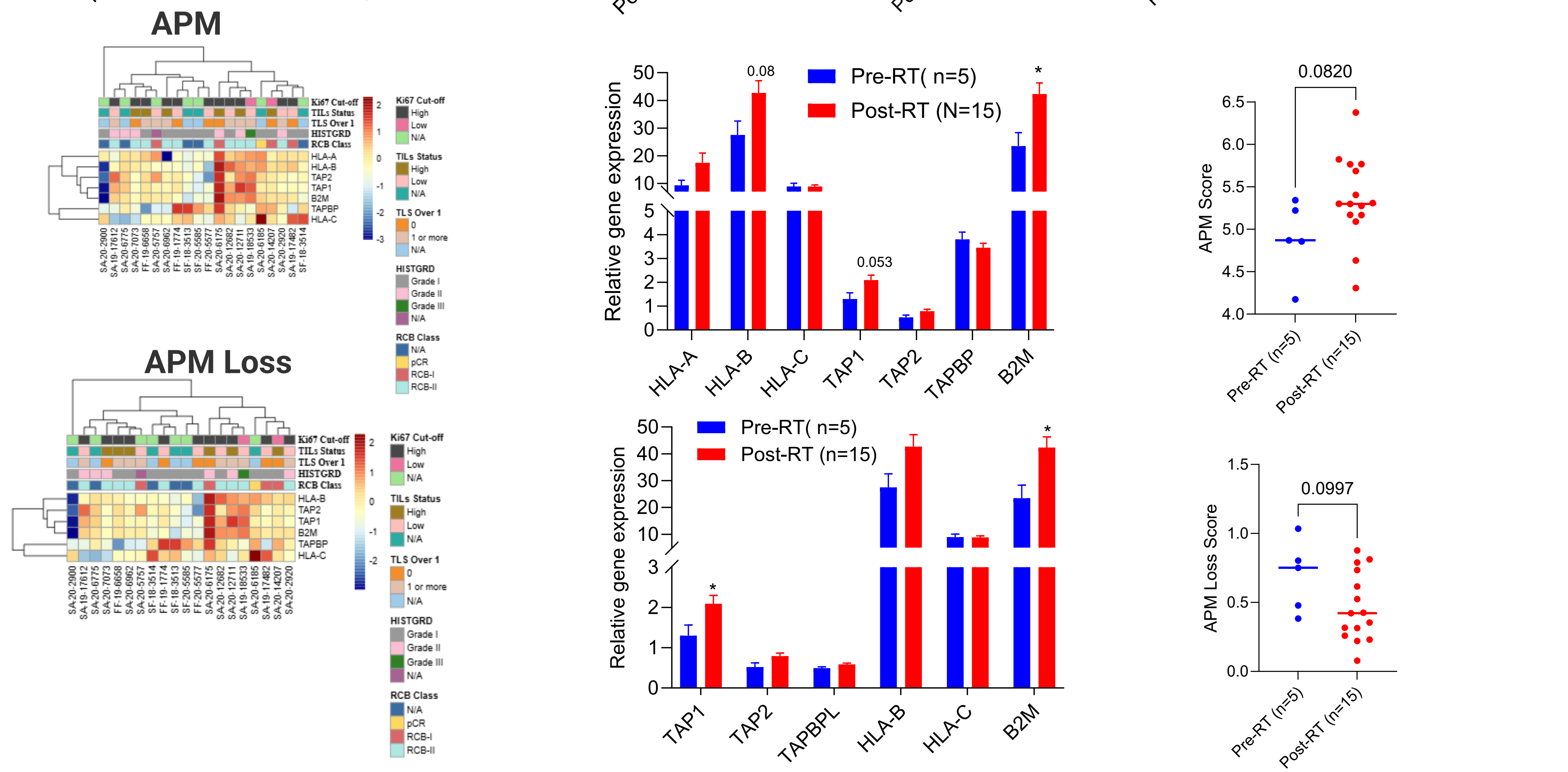

Supplement: Supplementary file 1 [file ijms-27-03227-s001.zip › S12.pdf]

Fig. S2

A

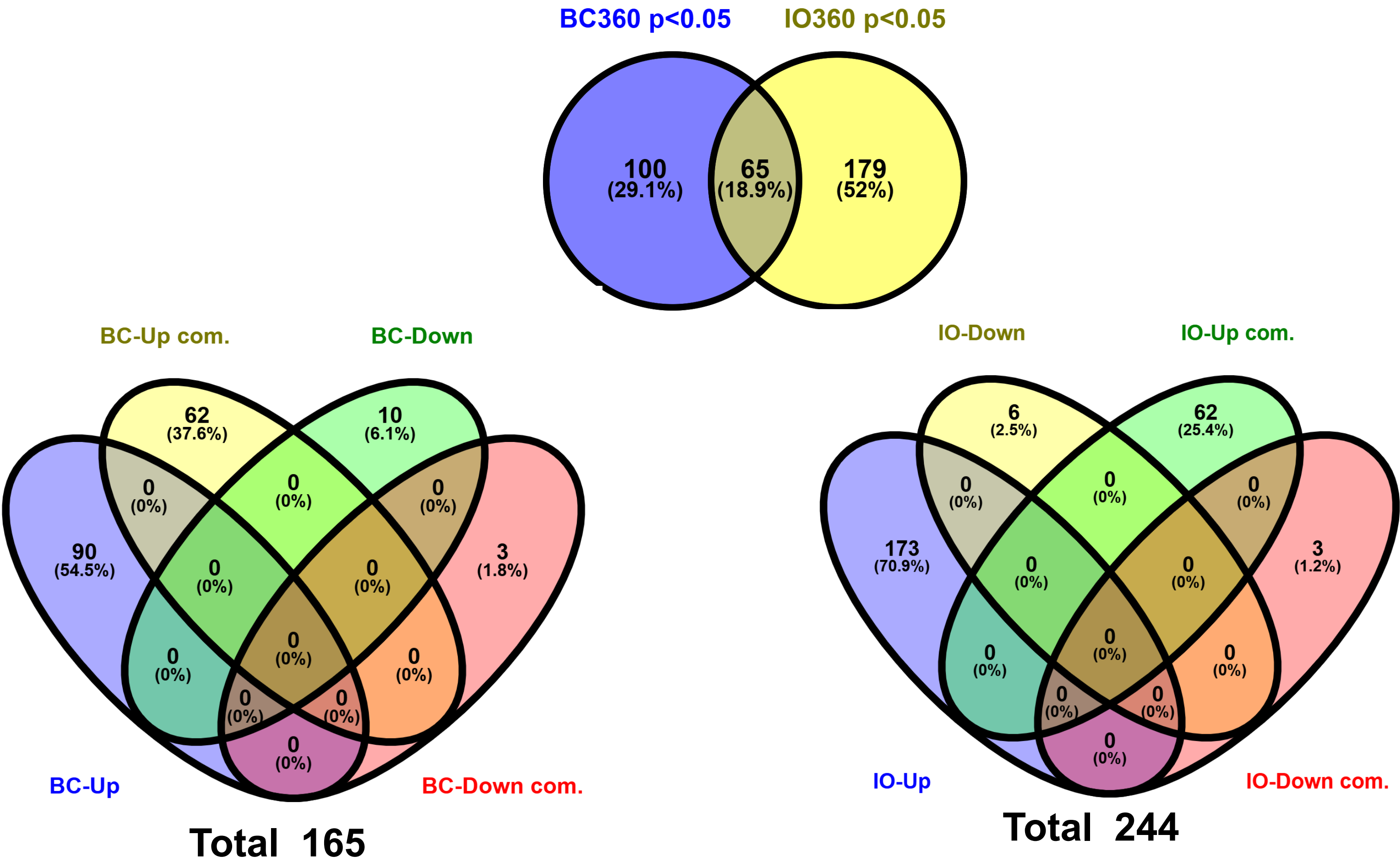

B

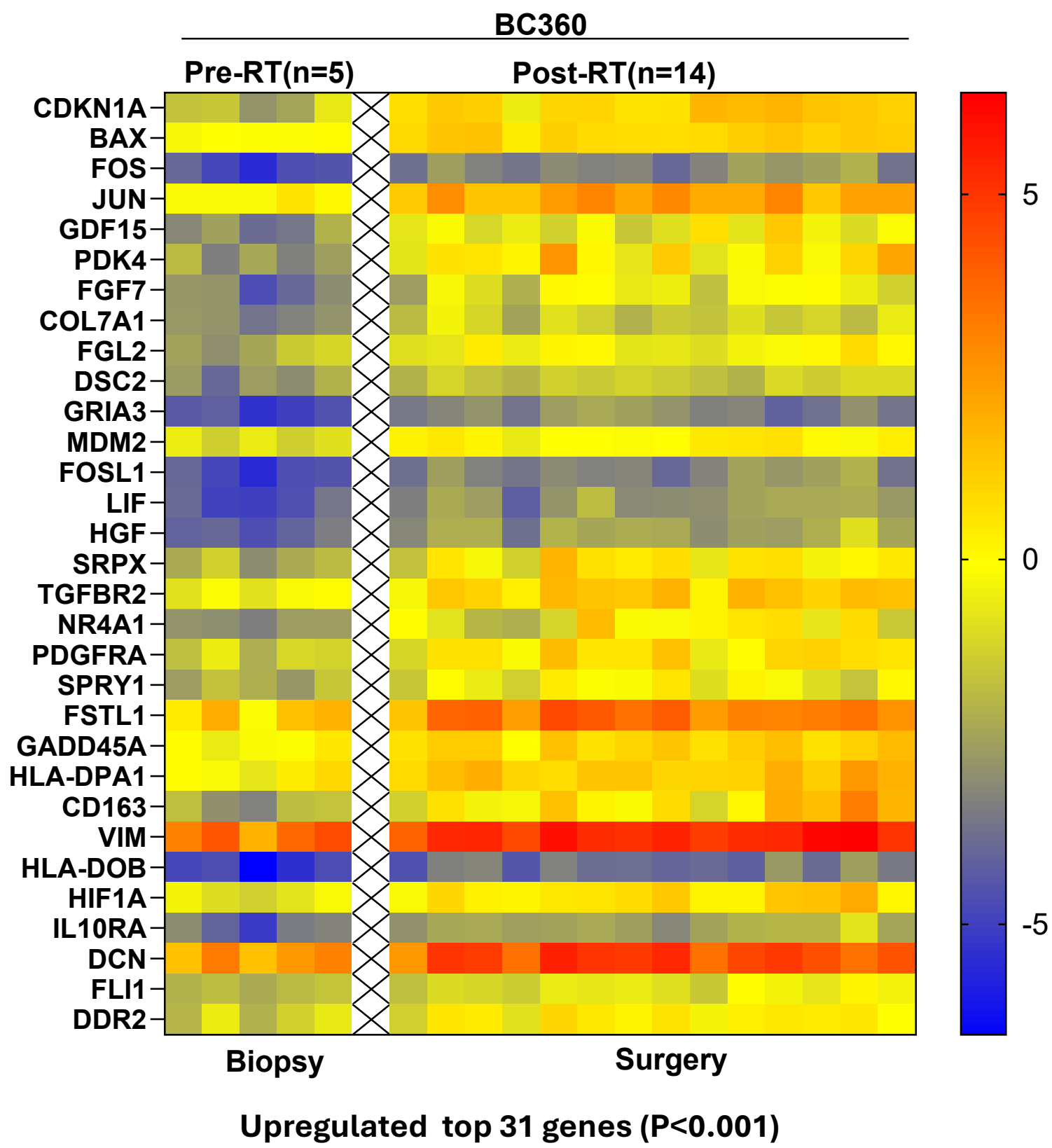

C

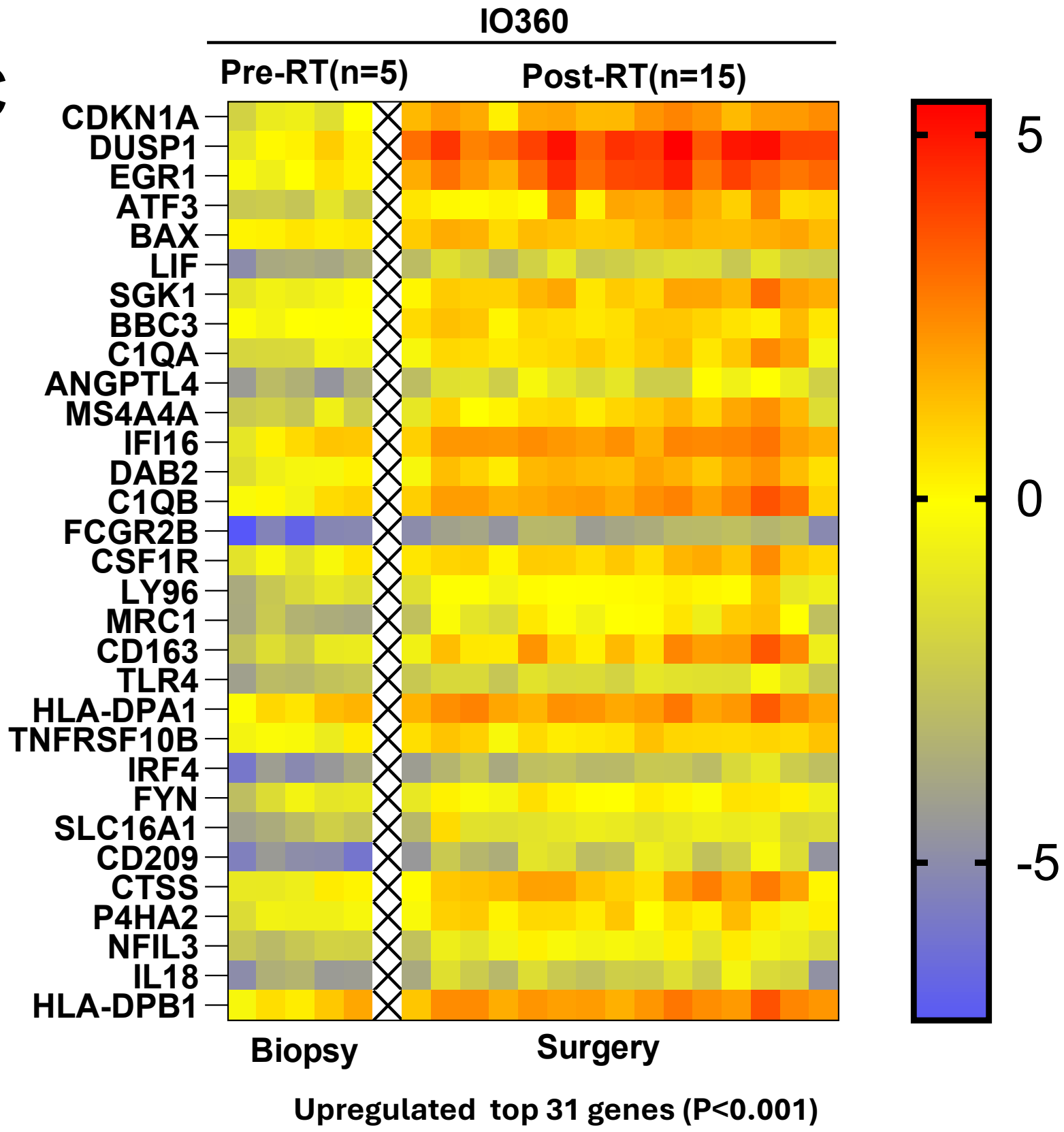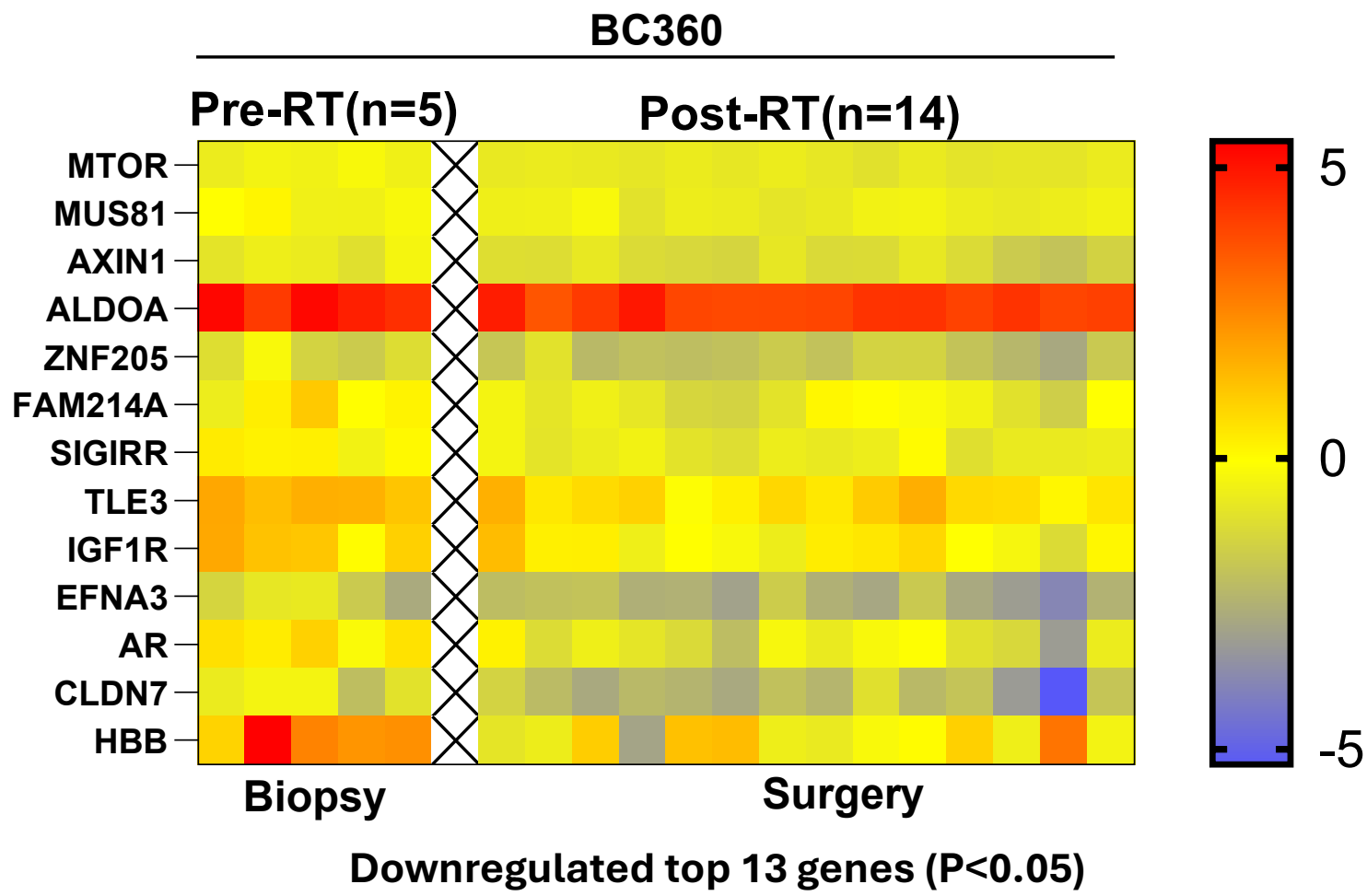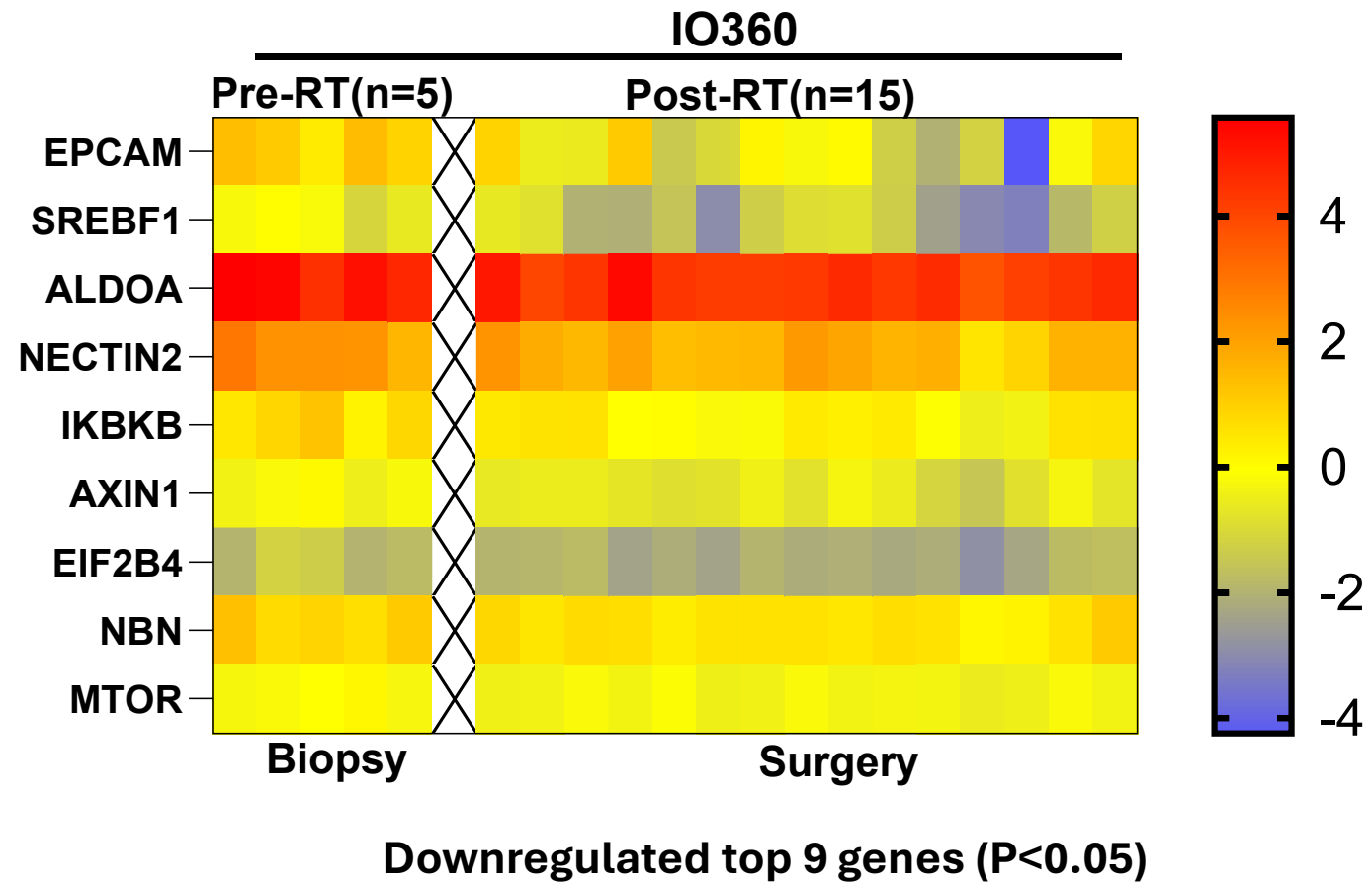

Supplement: Supplementary file 1 [file ijms-27-03227-s001.zip › S2.pdf]

**A**

# B

# HRD

## Genomic Risk

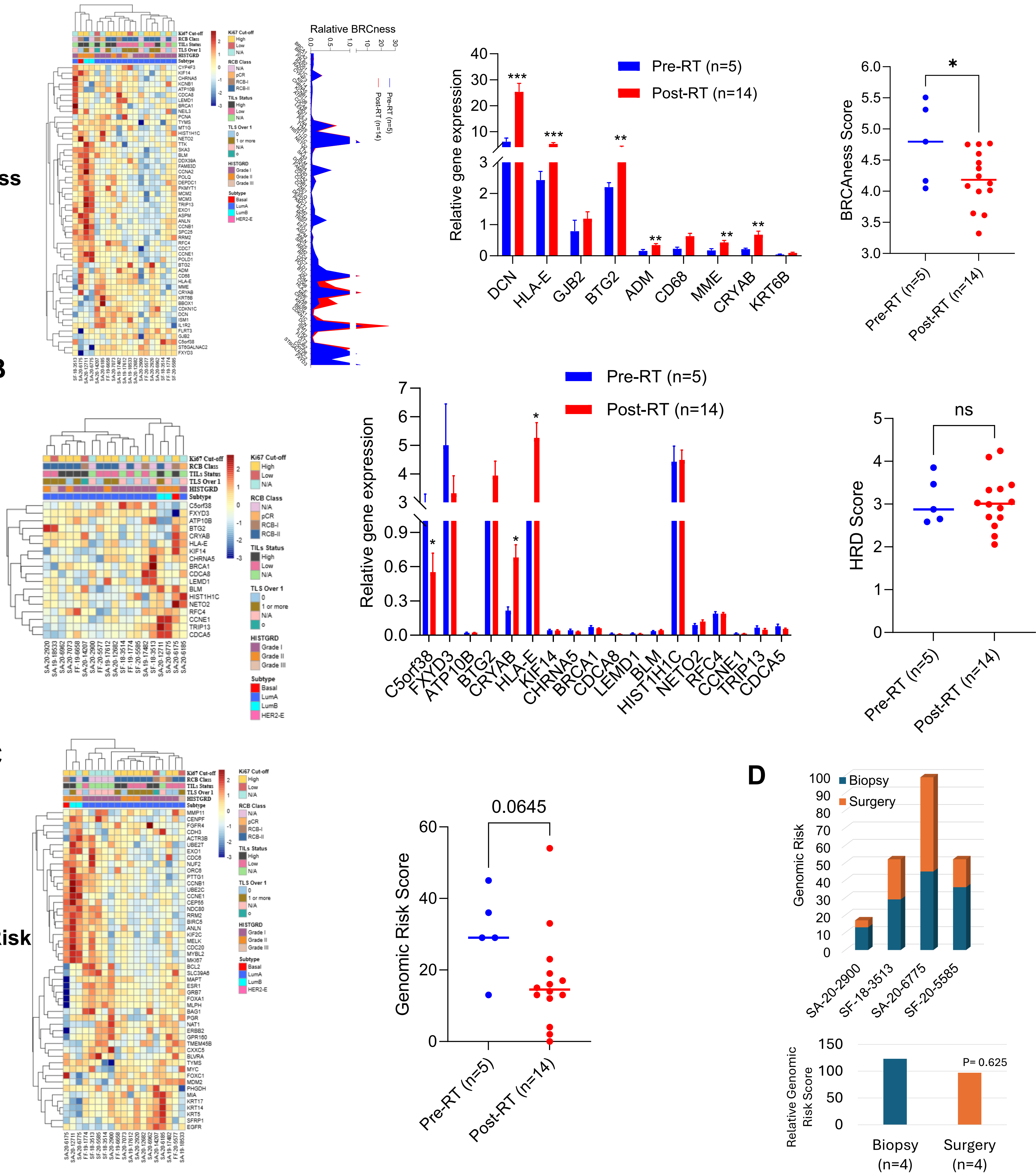

Supplement: Supplementary file 1 [file ijms-27-03227-s001.zip › S3.pdf]

Fig. S4

A

ER- Signaling

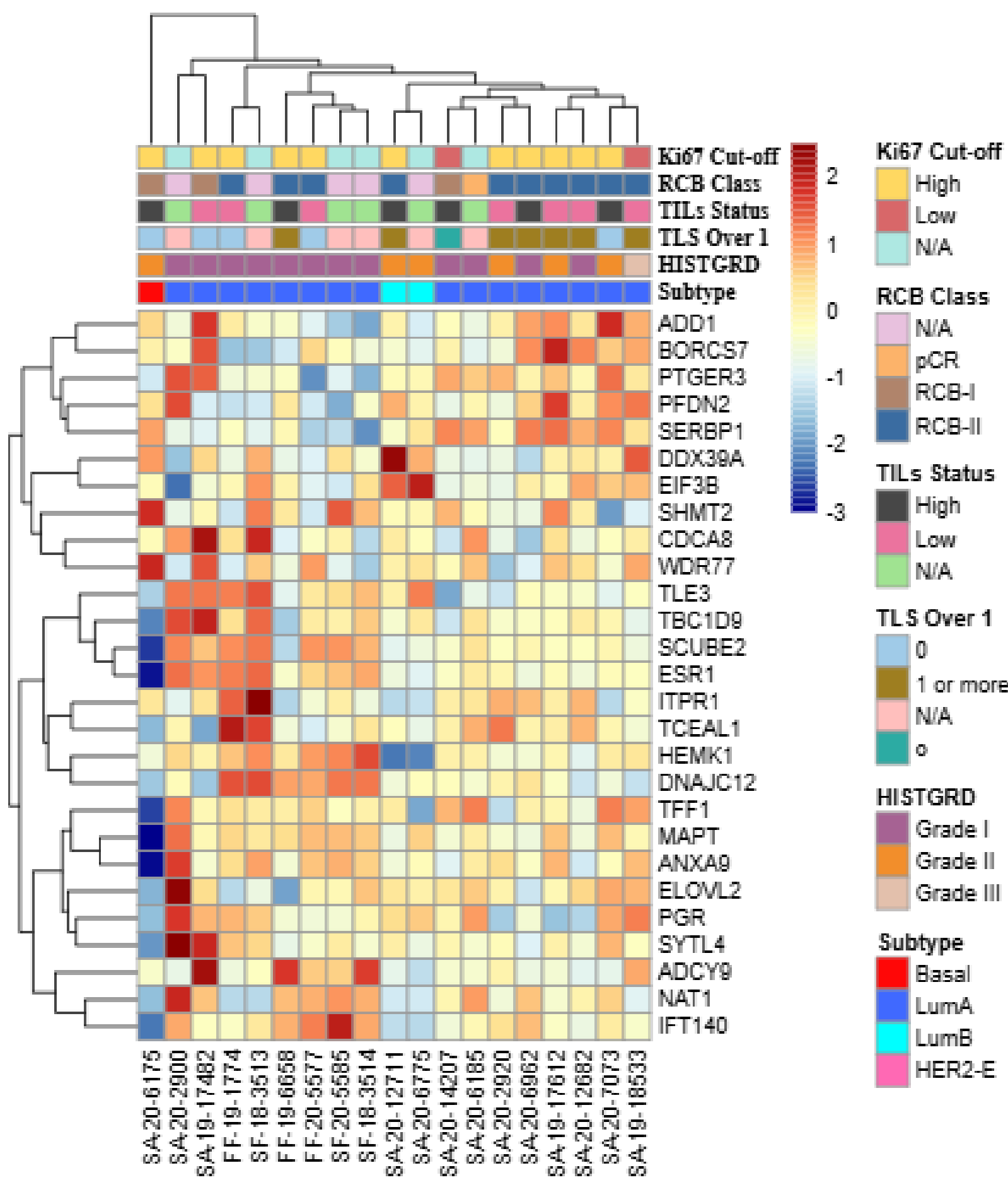

B

p53-related signature

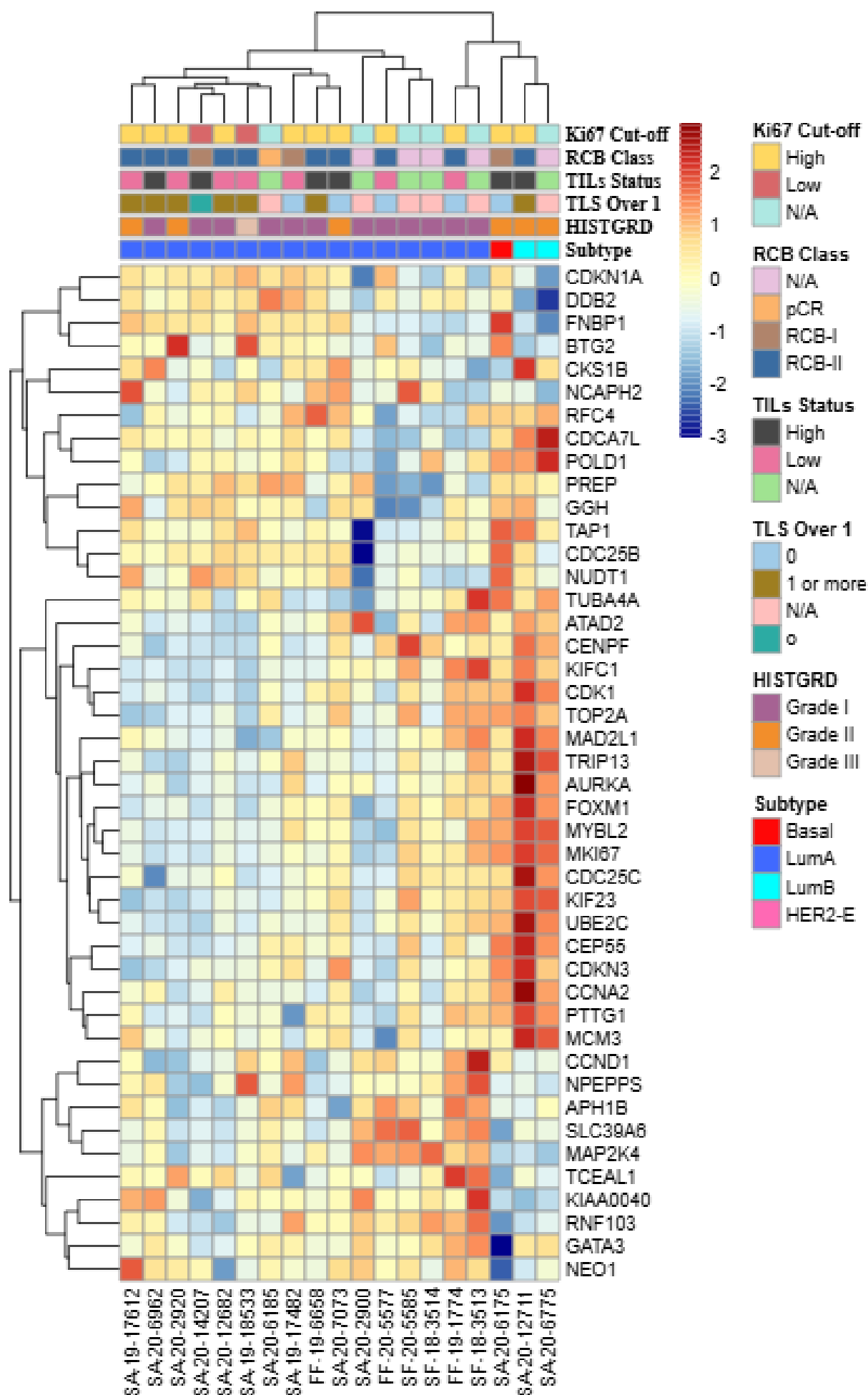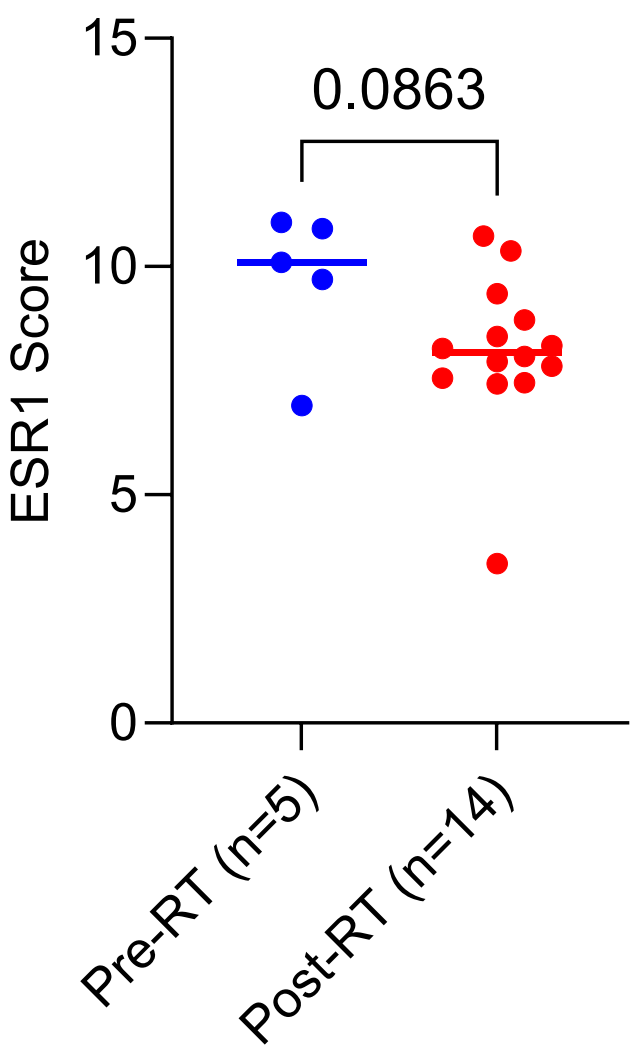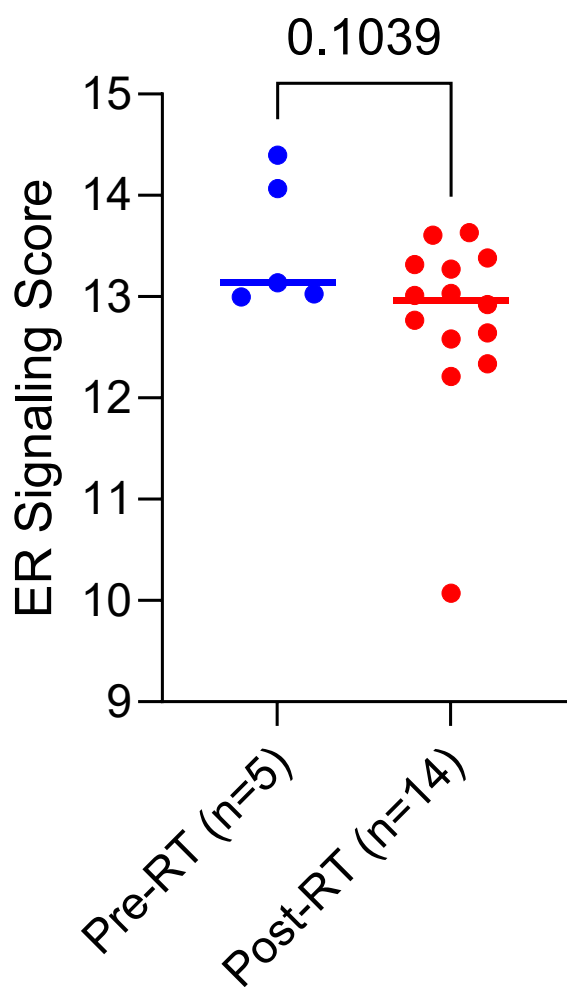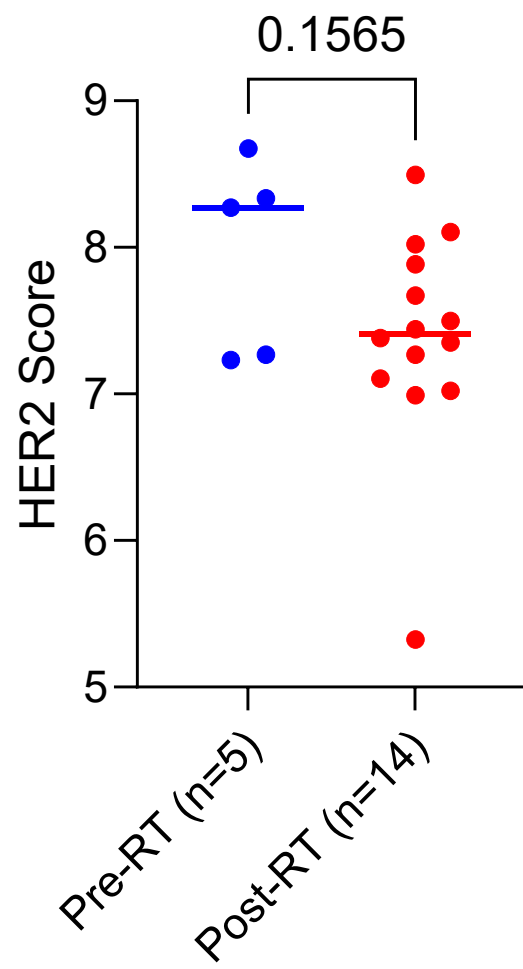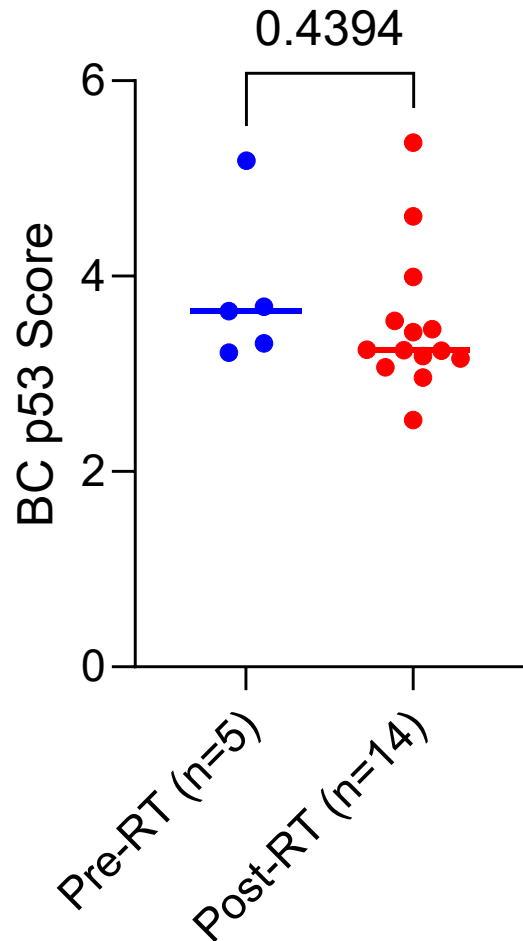

Supplement: Supplementary file 1 [file ijms-27-03227-s001.zip › S4.pdf]

Fig. S5

BC-360

A

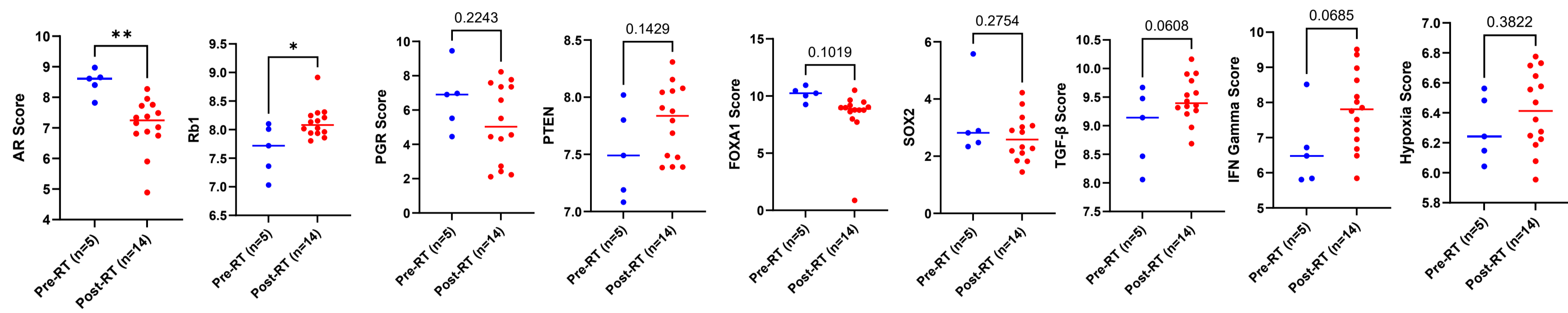

B

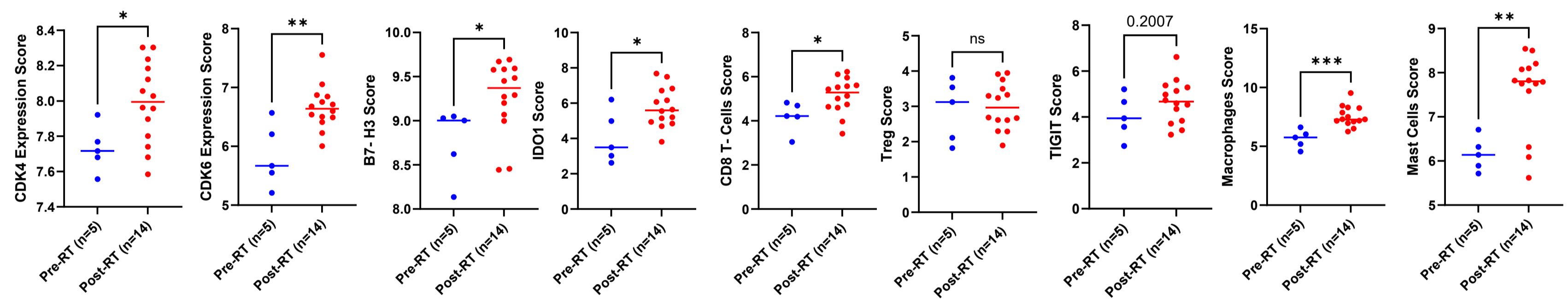

C

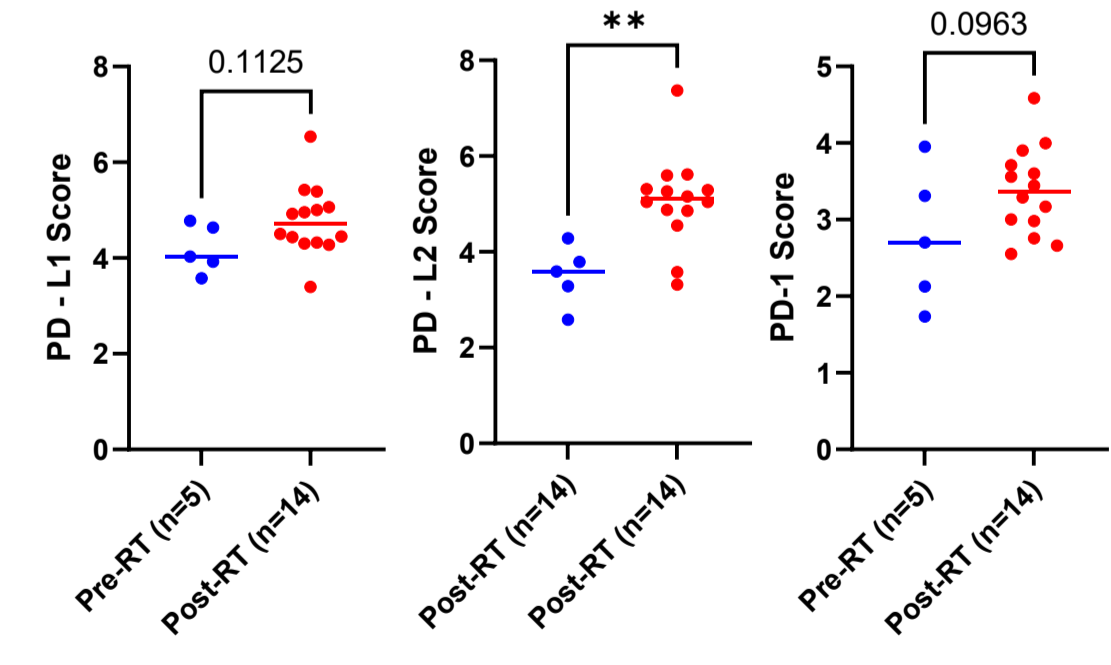

Supplement: Supplementary file 1 [file ijms-27-03227-s001.zip › S5.pdf]

Fig. S6

A

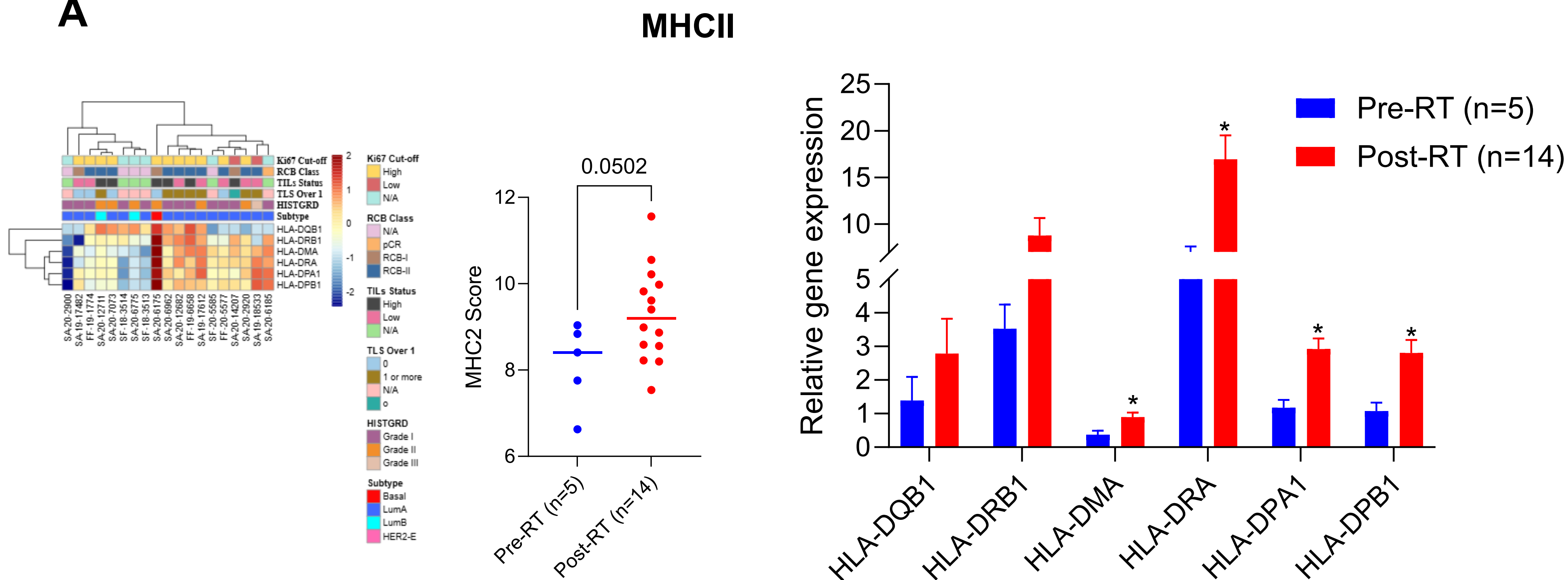

B

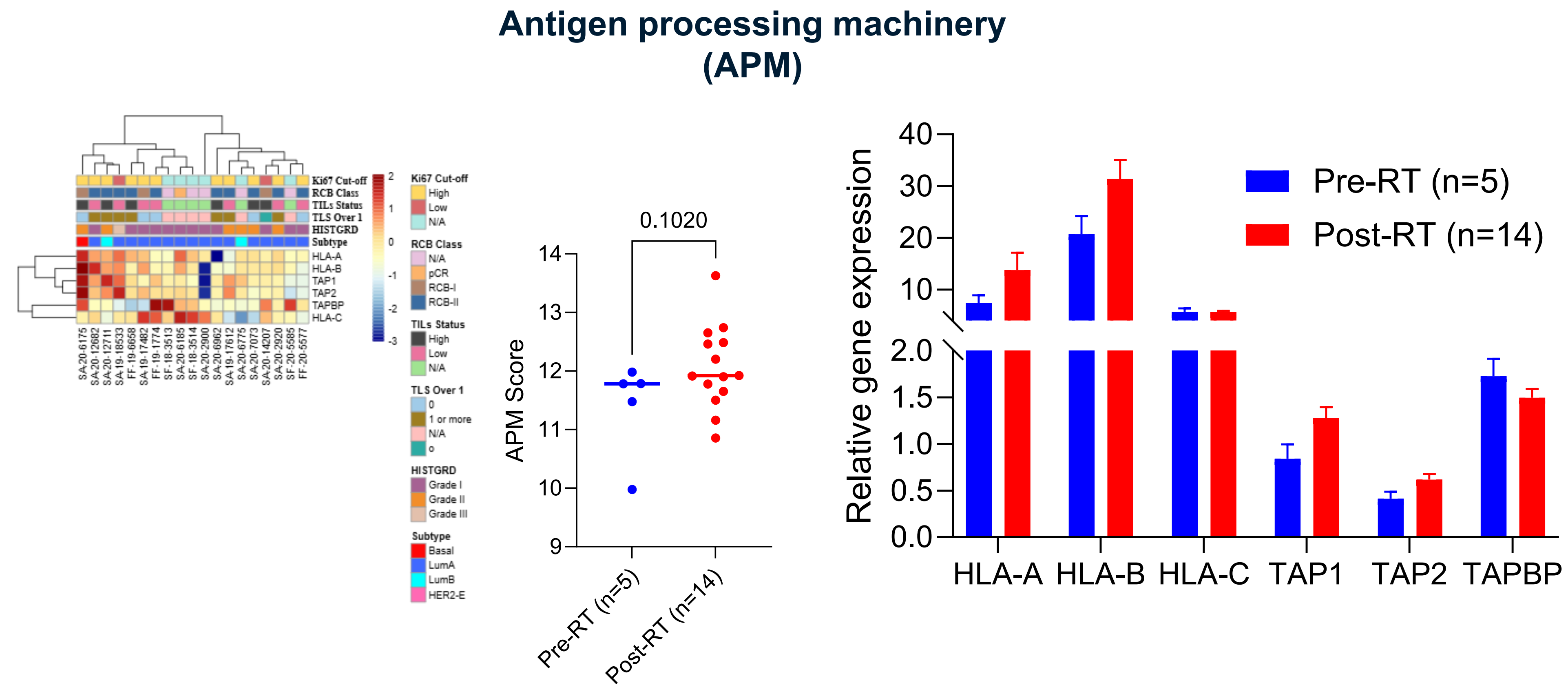

Supplement: Supplementary file 1 [file ijms-27-03227-s001.zip › S6.pdf]

**Fig. S8**

**IO360 TIS**

**A**

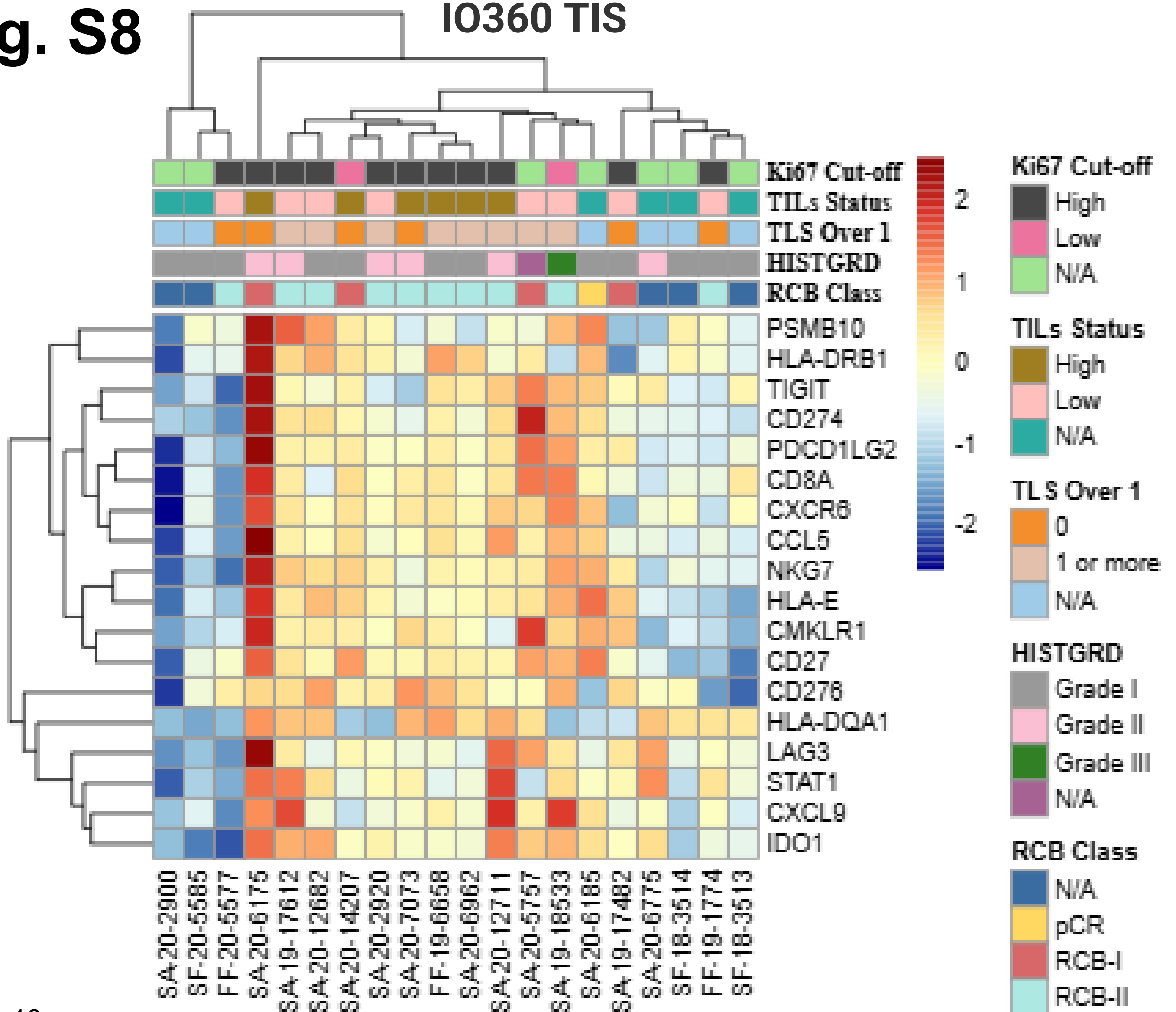

**B**

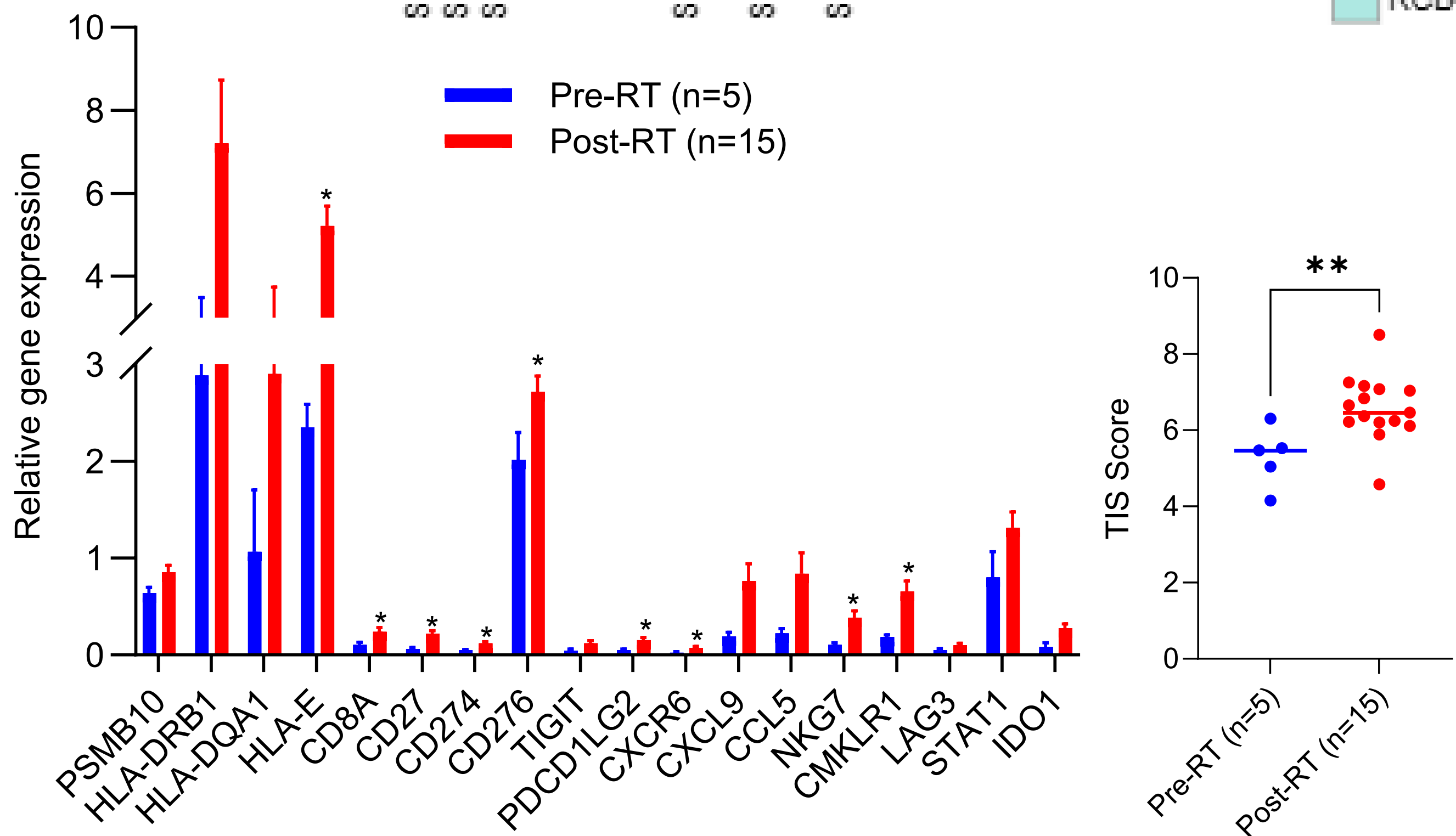

Supplement: Supplementary file 1 [file ijms-27-03227-s001.zip › S8.pdf]

Fig. S9

A

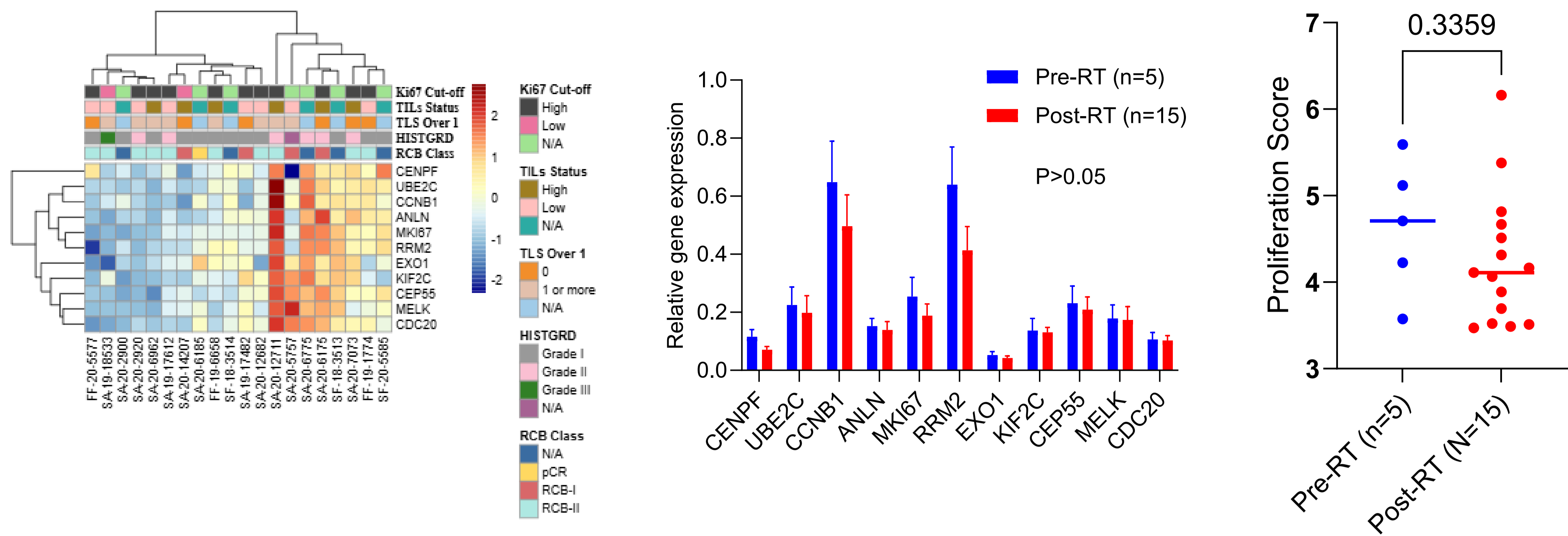

B

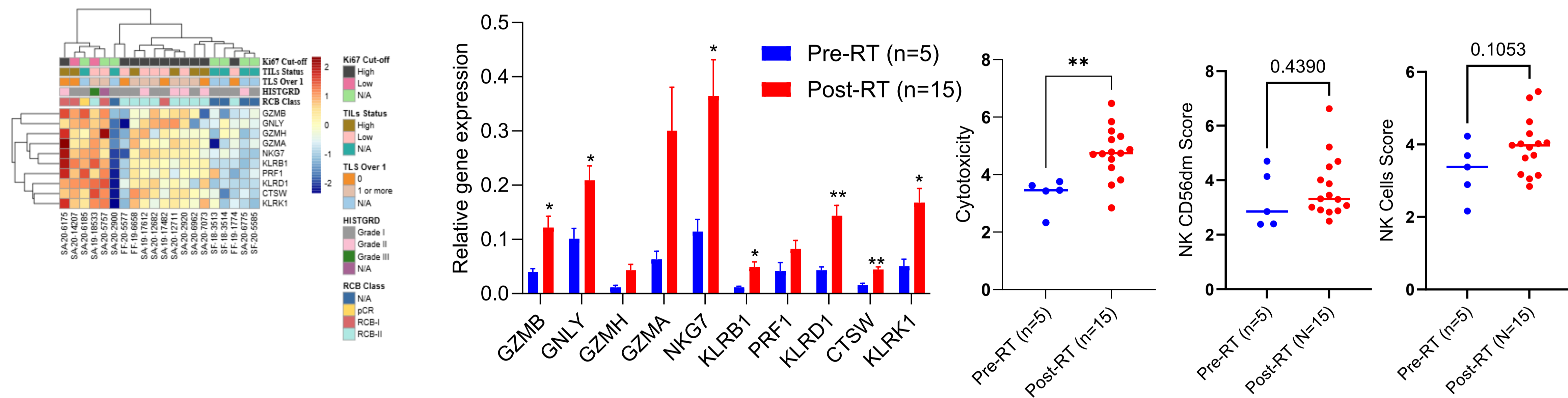

C

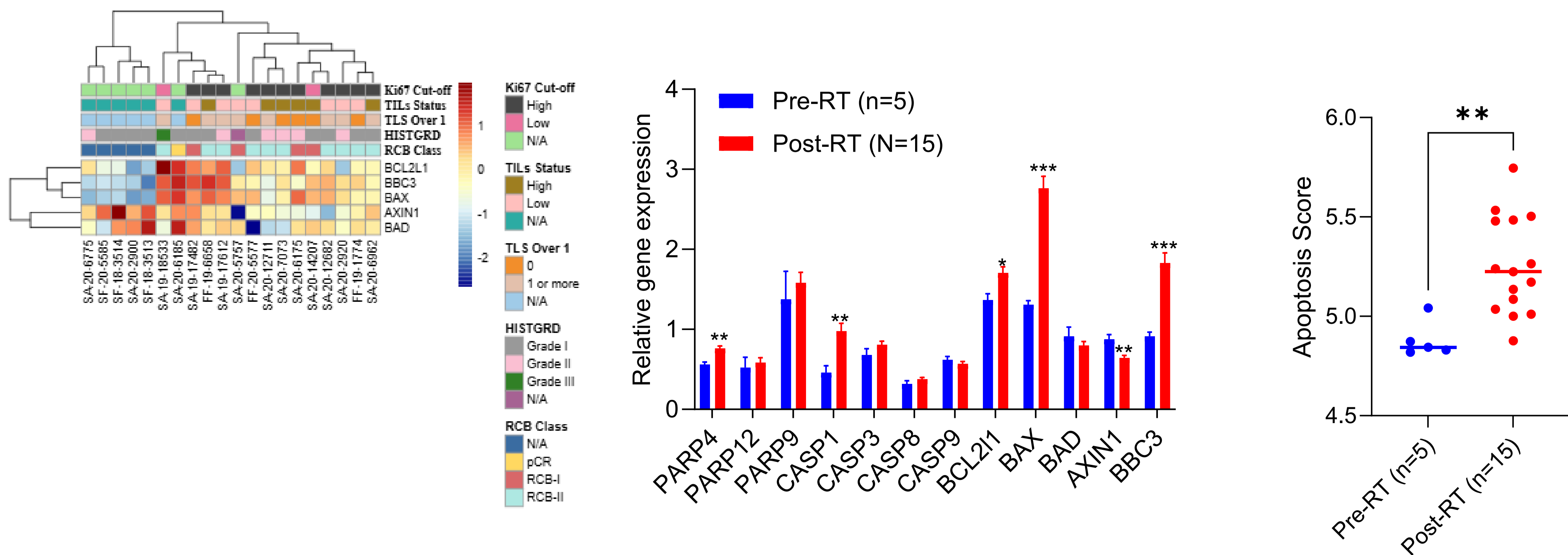

Supplement: Supplementary file 1 [file ijms-27-03227-s001.zip › S9.pdf]
