# Supplementary material for: Characterization of Oncogenic and Immunogenic Profiling in Patients with Breast Cancer Tumors After Radiation Therapy
Source: Int J Mol Sci. 2026 Apr 2;27(7):3227. doi: 10.3390/ijms27073227 (PMC13073414; doi:10.3390/ijms27073227)
Supplement: Supplementary file 1 [file S13.pdf › S13.pdf]

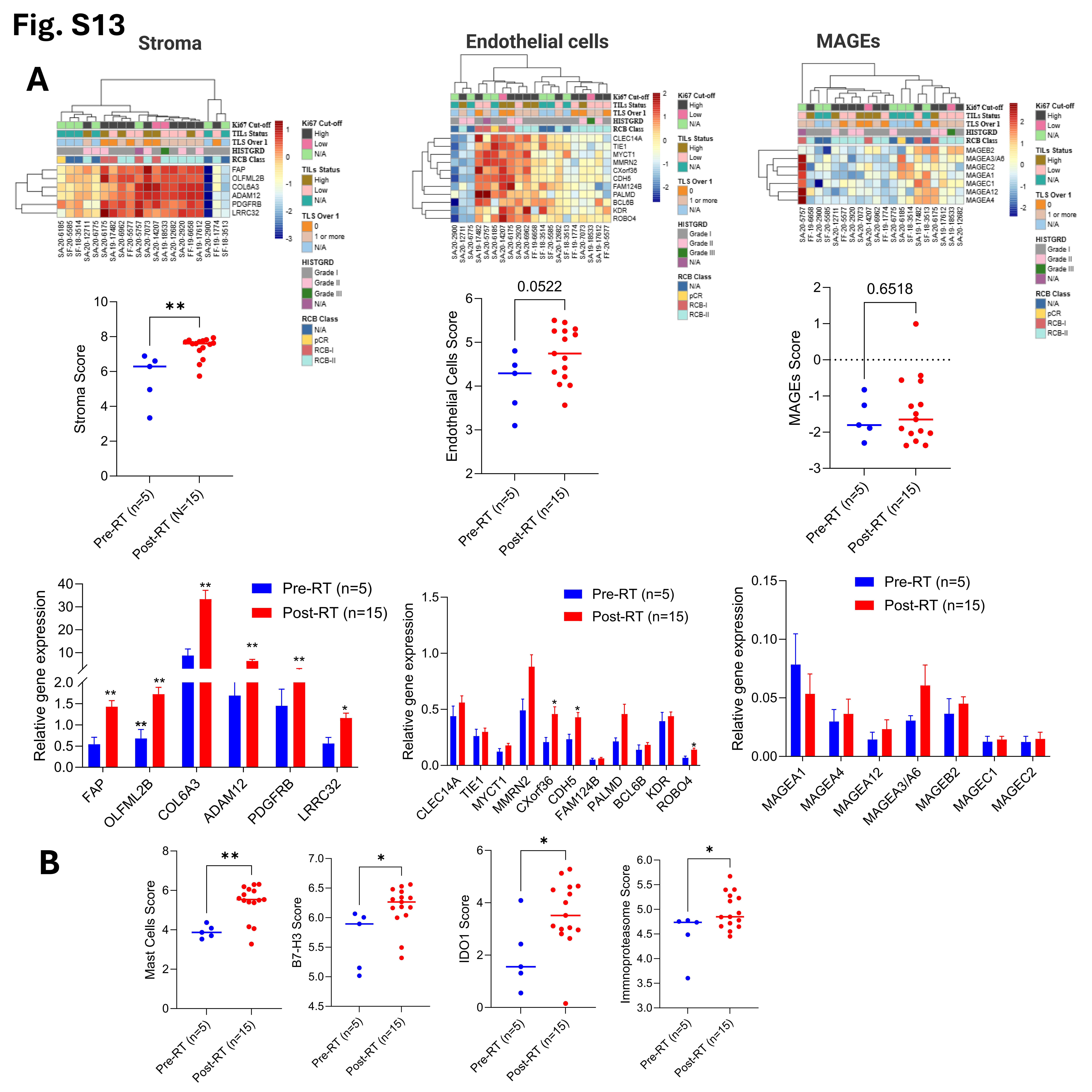

Fig. S13
A
Stroma
Endothelial cells
MAGEs
Ki67 Cut-off
TILs Status
TLS Over 1
HISTGRD
RCB Class
High
Low
N/A
0
1 or more
Grade I
Grade II
Grade III
pCR
RCB-I
RCB-II
FAP
OLFML2B
COL6A3
ADAM12
PDGFRB
LRRC32
CLEC14A
TIE1
MYCT1
MMRN2
CXorf36
CDH5
FAM124B
PALMD
BCL6B
KDR
ROBO4
MAGEB2
MAGEA3/A6
MAGEC2
MAGEA1
MAGEC1
MAGEA12
MAGEA4
Stroma Score
**
Pre-RT (n=5)
Post-RT (N=15)
Endothelial Cells Score
0.0522
Post-RT (n=15)
MAGEs Score
0.6518
Relative gene expression
Pre-RT (n=5)
Post-RT (n=15)
B
Mast Cells Score
B7-H3 Score
IDO1 Score
Immnoproteasome Score
